# Trends in the quality and cost of inpatient surgical procedures in the United States, 2002–2015

**DOI:** 10.1371/journal.pone.0259011

**Published:** 2021-11-03

**Authors:** Ning Ning, Alex Haynes, John Romley

**Affiliations:** 1 Department of Pharmaceutical and Health Economics, USC School of Pharmacy, University of Southern California, Los Angeles, CA, United States of America; 2 Dell Medical School, University of Texas at Austin, Austin, TX, United States of America; 3 Public Policy, USC Price School of Public Policy, University of Southern California, Los Angeles, CA, United States of America; 4 Schaeffer Center for Health Policy and Economics, University of Southern California, Los Angeles, CA, United States of America; Ohio State University Wexner Medical Center Department of Surgery, UNITED STATES

## Abstract

**Objectives:**

This study documents trends in risk-adjusted quality and cost for a variety of inpatient surgical procedures among Medicare beneficiaries from 2002 through 2015, which can provide valuable insight on future strategies to improve public health and health care.

**Methods:**

We focused on 11 classes of inpatient surgery, defined by the Agency for Health Research and Quality’s (AHRQ’s) Clinical Classification System. The surgical classes studied included a wide range of surgeries, including tracheostomy, heart valve procedures, colorectal resection, and wound debridement, among others. For each surgical class, we assessed trends in treatment costs and quality outcomes, as defined by 30-day survival without unplanned readmissions, among Medicare beneficiaries receiving these procedures during hospital stays. Quality and costs were adjusted for patient severity based on demographics, comorbidities, and community context. We also explored surgical innovations of these 11 classes of inpatient surgery from 2002–2015.

**Results:**

We found significant improvements in quality for 7 surgical classes, ranging from 0.08% (percutaneous transluminal coronary angioplasty) to 0.74% (heart valve procedures) per year. Changes in cost varied by surgery, the significant decrease in cost ranged from -2.59% (tracheostomy) to -0.34% (colorectal resection) per year. Treatment innovation occurred with respect to surgical procedures utilized for heart valve procedures and colorectal resection, which may be associated with the decrease in surgical cost.

**Conclusions:**

Our results suggest that there was significant quality improvement for 7 surgery categories over the 14-year study period. Costs decreased significantly for 6 surgery categories, and increased significantly for 3 other categories.

## Introduction

Controlling healthcare spending while simultaneously improving quality of service has been an ongoing effort in the US. By the end of 2014, healthcare spending in the US reached $3.0 trillion [1], corresponding to a 5.3% increase in that year alone [1–3]. Some have suggested, however, that a substantial proportion of healthcare spending brings little or no value [4]. The Institute of Medicine stated that “the only sensible way to restrain costs is to enhance the value of the healthcare system, thus extracting more benefit from the dollars spent.” [5].

Inpatient surgical care constitutes a large proportion of spending in hospitals. Over the past several decades, the long-term changes in inpatient surgical quality and cost have been affected by a variety of factors, including physician skill, surgical knowledge, price of supplies, policy change, technology innovation, healthcare condition, etc. Improvements in physician skills, surgical knowledge, technology may have made surgeries safer, but possibly more expensive and complicated in some circumstances [6]. Policies introduced by the Centers for Medicare and Medicaid Services (CMS), such as the Hospital Value-Based Purchasing Program and Hospital Readmissions Reduction Program (HRRP), may strive to control hospital inpatient admissions, unplanned readmissions, and unnecessary costs [7–9], but at the same time may also encourage development of new technologies that increase surgical costs. Innovative surgical procedures may result in better surgical care and fewer readmissions, but they may also increase use of surgery as a treatment option for patients in more critical health conditions, potentially leading to decreased survival rates and increased readmissions. Hence, the real long-term changes in inpatient surgical quality and cost over time remain unknown. In this study, we aim to evaluate the value of inpatient surgical care by studying long-term changes in cost and quality outcomes, relative to patient illness severity and demographic information.

Numerous published studies have assessed the quality and cost of surgical care, many of which use mortality rates to quantify surgical quality. Some studies also use decrease in short-term readmission rates as an indicator of improved quality, and most of these focus primarily on specific treatments, hospitals, or reforms [10–14]. Additionally, few studies have analyzed the underlying factors that affect readmission rates, such as specific characteristics of patients or hospitals [15, 16]. For reports assessing surgical cost, most utilized cost-effectiveness analysis [17, 18], which did not factor in the trends in surgical cost and quality over time.

Only a limited number of studies have analyzed long-term changes in quality (mortality and readmission rates) and cost of healthcare services. One report found that between 1996 and 2006, mortality rates following inpatient surgery improved significantly for only one of 11 surgery types [6]. However, this study did not measure surgical readmissions or costs. Another study assessed productivity, defined based on mortality and readmission rates, in Medicare patients treated for heart attack, heart failure, and pneumonia from 2002–2011 [19]. Here, however, they did not measure long-term survival rates, unplanned readmission rates, or cost; nor did they assess inpatient surgical care. Critically, to our knowledge, there are no published studies measuring longer-term patterns in cost and quality for a wide range of inpatient surgeries at the national level in the U.S., particularly those that incorporate mortality and unplanned readmission rates in their analyses. We assess trends for a group of previously studied inpatient surgeries [6], but over a more recent period (2002–2015) and also addressing costs.

In this study, we sought to answer two questions: What are the long-term patterns of surgery quality and cost? Can variation in quality and cost of surgical care be fully explained by changes in patient demographics and illness severity? We also sought to explore innovations in surgical care that have been implemented from 2002–2015. We hope that by answering these questions and studying long-term trends in survival rates, unplanned readmissions, and cost, we can provide meaningful insight on the assessment of inpatient surgery performance and introduction of quality improvement programs.

## Methods

### Data

Our primary data source was the Medicare 20% Part A and Part B claims data from 2002–2015. These claims data include patient diagnosis codes, inpatient service procedure codes, non-institutional service Healthcare Common Procedure Coding System (HCPCS) codes, dates of service, hospital discharge dates, provider identification, and service charges. The research-identifiable version of the data also reports patient demographic information, including zip codes and death dates. We further integrated information from the 2010 US Census to add community-level characteristics to the data. The University of Southern California Institutional Review Board approved this study (UP-14-00636). Consent was not obtained because the data were analyzed anonymously.

### Surgeries

We included specific categories of surgeries based on the International Classification of Diseases, Ninth Revision, Clinical Modification (ICD-9-CM) codes, as defined and selected by five independent surgeons in a previous study [6]. These categories include both non-operating room and operating room procedures that involve “incision, excision, suturing, and manipulation of tissue, usually with regional or general anesthesia or profound sedation to control pain” [6]. Similar interventions were grouped together into 11 categories based on the Clinical Classification System (CCS) of the Agency for Healthcare Research and Quality (AHRQ) [6].

### Sample

We first identified patients who underwent at least one of the selected inpatient surgical procedures from 2002 through September 2015 (beginning October, 2015, CMS mandated the use of an updated coding scheme, specifically, ICD-10-CM). Because inpatient admission type was used as one of the proxies for illness severity, patients whose inpatient admission types were missing were excluded. Finally, we limited our cohorts to patients enrolled in fee-for-service (FFS) Medicare within this study period because we do not believe that inpatient services can be consistently identified for patients enrolled in Medicare HMO.

### Statistical analysis

To quantify surgical quality, we first identified whether a hospitalization was a high-quality stay. This was defined as a stay in which a patient survived for at least 30 days after his/her initial hospital admission and was not readmitted with an unplanned readmission within 30 days of discharge. Methodology for identifying unplanned readmissions was provided by the CMS [20, 21].

To quantify surgical cost, we summed the costs of inpatient stays and physician services based on the Medicare Part A and Part B claims. To calculate the cost of inpatient stays, we first used the cost-to-charge ratio, which hospitals report to the CMS, to estimate inpatient costs based on total Medicare hospital charges [19, 22]. We then adjusted the labor-related portion of the base payment rate in the Inpatient Prospective Patient System (IPPS), using the hospital wage index to account for geographic differences in labor prices [19]. Next, we adjusted for inflation by converting costs to 2015 US dollars using the CMS inpatient market basket of the prospective payment system. In order to incorporate non-institutional costs, we selected all Part B claims billed by physicians during each inpatient stay. Finally, we converted costs of non-institutional claims to 2015 US dollars using the Medicare economic index and economy-wide private nonfarm business multifactor productivity from the Physician Fee Schedule.

We used logistic regression to evaluate changes in surgical quality and multivariate linear models to assess changes in cost over the study period. We first regressed the outcomes on a group of year indicators using the whole sample from 2002–2015, in order to estimate rates of high-quality stays and surgical cost for each year. We then used only the data from 2002 and 2015 to regress the outcomes on one year indicator and calculate annualized growth rates of quality and cost, thereby identifying cumulative changes that occurred from 2002–2015. In additional analysis, we decomposed changes in overall quality (survival without an unplanned readmission) into changes in survival and readmission avoidance. All regressions were performed at the beneficiary level, and standard errors were clustered at the hospital level to account for correlations in the residuals.

To address the potential confounding effect of changes in patient illness severity, we adjusted for admission types and Charlson comorbidities, as indicated in the hospital admission/discharge records [19]. In addition, we adjusted for patient demographics and socio-demographic information, including age, gender, race/ethnicity, area poverty rate, percentage of elderly residents with disabilities, percentage of residents with a high level of education, and other factors. We also defined and controlled for hospital teaching status [19], with teaching hospitals defined as those with a resident-to-bed ratio >0.6. We then performed regressions with these control variables. Additional analyses interacted patient age with the indicator variable for the year 2015, and excluded admissions whose type was coded as “newborn.”

We also briefly examined treatment innovation during the study period. Surgical innovations were defined as clinically distinct procedures that were nonexistent in 2002. These were identified using their five-digit ICD-9-CM procedure codes. We also searched the selected ICD-9-CM procedures, and excluded the procedures which had already existed in the market but which received revised procedure codes in later years. We calculated frequencies of novel procedures within the different surgery categories for each calendar year and compared the timing of changes in surgical cost and quality with the timing of change in market shares of surgical innovations.

Because the CMS define unplanned readmissions based on whether patients are readmitted within 30 days after hospitalization discharges, there is a possibility that some beneficiaries did not have readmissions because they died within this 30-day period. To address this possibility, we redefined the quality measure in a sensitivity analysis, in which patients were considered to have a high-quality stay only if they survived for 30 days after initial hospital discharge without any unplanned readmissions. In this sensitivity analysis, the cost measure was also redefined as mean cost per day during hospitalization. Finally, for patients with heart attack only, we adjusted for specific anatomical locations of the heart attack, e.g., inferolateral wall and inferoposterior wall, and predicted likelihood of death during the hospital stay, together with the control variables described above.

## Results

Patient samples for the 11 surgery categories range in size from 5,418 to 526,351 patients over the 14-year study period, with nine categories containing a sample size >40,000 patients (Table 1). Summary statistics of key variables are listed in S1 Table.

**Table 1:** The 11 surgery categories and corresponding sample sizes, 2002–2015.

CCS Category	Surgery Type	Number of Cases	Number of Hospitals
CCS 34	Tracheostomy	46,689	2,716
CCS 43	Heart Valve Procedures	111,333	1,277
CCS 44	Coronary Artery Bypass Grafting (CABG)	196,061	1,303
CCS 45	Percutaneous Transluminal Coronary Angioplasty (PTCA)	526,351	1,909
CCS 71	Gastrostomy	100,026	3,405
CCS 73	Ileostomy and Other Enterostomy	5,418	1,814
CCS 75	Small Bowel Resection	46,067	3,138
CCS 78	Colorectal Resection	231,575	3,546
CCS 89	Exploratory Laparotomy	7,413	2,164
CCS 90	Excision, Lysis Peritoneal Adhesions	51,373	3,215
CCS 169	Debridement of Wound	110,206	3,581
	**Total**	**1,432,512**	**28,068**

Abbreviation: CCS, Clinical Classification System. It is developed at the Agency for Healthcare Research and Quality (AHRQ).

**Fig 1:**
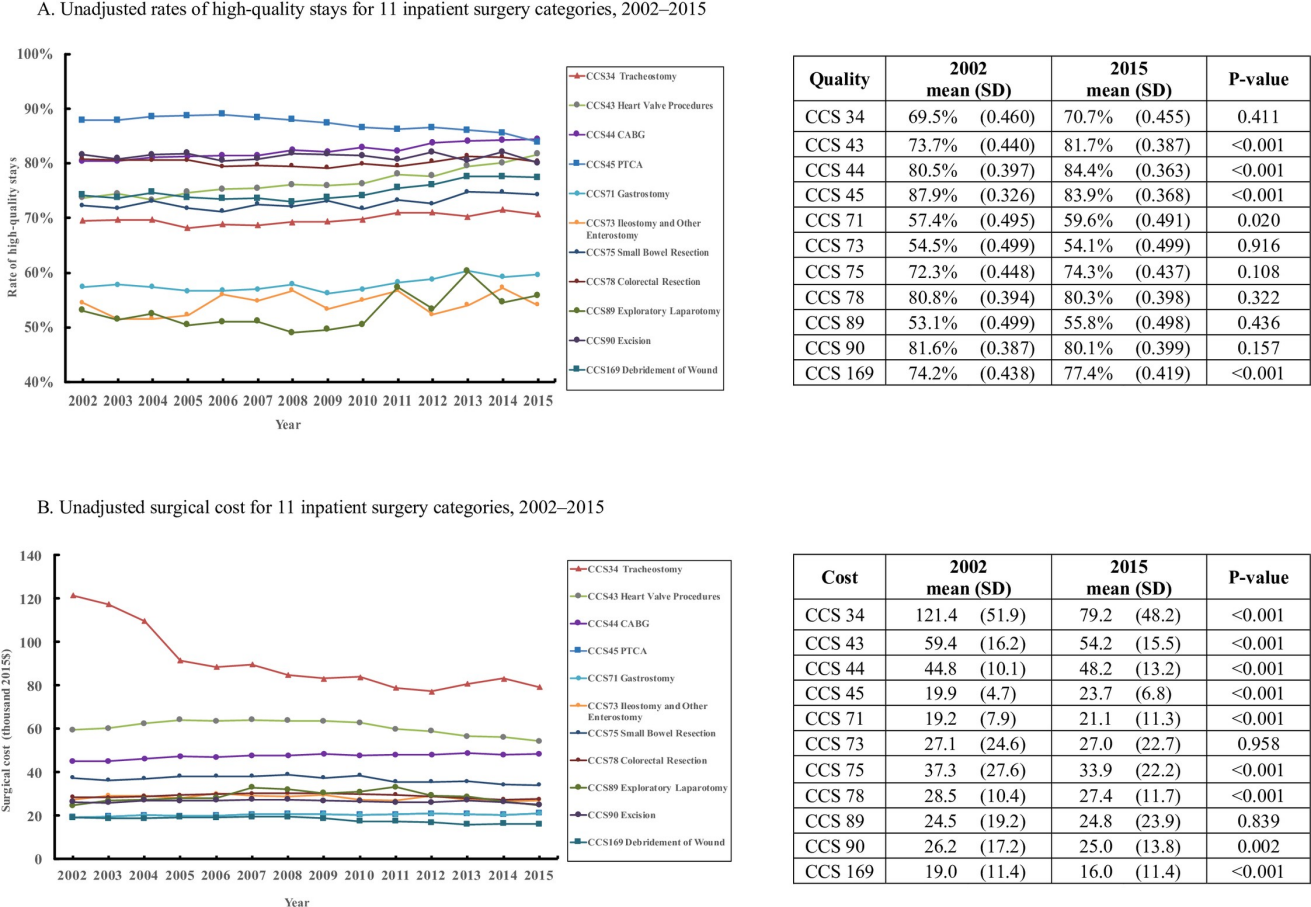
Unadjusted rates of high-quality stays and cost for 11 inpatient surgery categories, 2002–2015. (A) Unadjusted rates of high-quality stays for 11 inpatient surgery categories, 2002–2015. (B) Unadjusted surgical cost for 11 inpatient surgery categories, 2002–2015.

Unadjusted surgical quality and cost are presented in Fig 1. We found that trends in quality and cost changed smoothly and were varied by surgery. There was a general improvement in quality for 7 surgery categories, whereas a general decrease in cost for 7 surgery categories (Fig 1). By the end of 2015, unadjusted quality of 4 surgical categories were significantly higher than 2002, whereas unadjusted cost of 6 surgical categories were significantly lower than 2002 (Fig 1).

**Fig 2:**
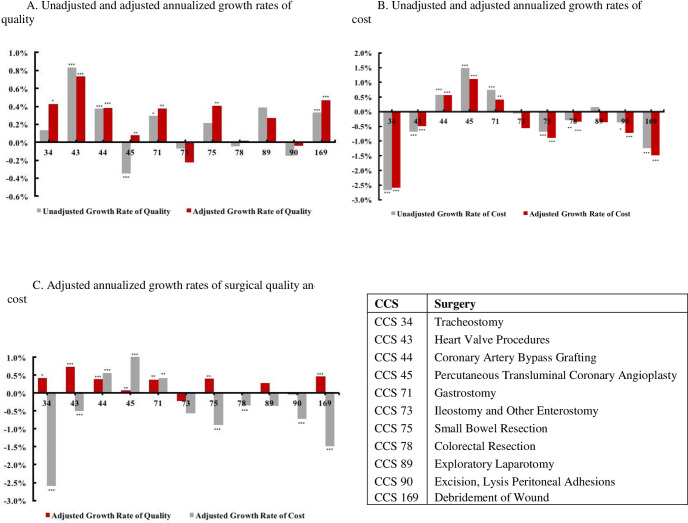
Unadjusted and adjusted annualized growth rates of quality and cost for 11 surgery categories, 2002–2015. (A) Unadjusted and adjusted annualized growth rates of quality. (B) Unadjusted and adjusted annualized growth rates of cost. (C) Adjusted annualized growth rates of surgical quality and cost.

When only data from 2002 and 2015 are considered, unadjusted logistic regressions reveal a significant decrease in quality for PTCA and significant increases in quality for heart valve procedures, CABG, gastrostomy, and debridement of wound (*P*<0.05 for each category). No significant changes in unadjusted quality were observed for the other six categories, although tracheostomy, small bowel resection, and exploratory laparotomy showed some slight increases in quality. Unadjusted annualized quality growth rates ranged from -0.35% to 0.83% per year for PTCA and heart valve procedures, respectively (Fig 2A).

Unadjusted growth in cost based only on data from 2002 and 2015 was negative for tracheostomy, heart valve procedures, small bowel resection, colorectal resection, excision/lysis peritoneal adhesions, and debridement of wound (*P*<0.05 for each category), indicating significant cost reductions for those procedures (Fig 2B). Conversely, significant increases in unadjusted costs were measured for CABG, PTCA, and gastrostomy (*P*<0.05 for each category). Among the 11 categories, tracheostomy displayed the largest decrease in cost (-2.67% per year), and PTCA showed the largest increase in unadjusted cost (1.49% per year).

After adjusting for patient demographics, illness severity, hospital characteristics, and social characteristics (Fig 2A), quality increased significantly for 7 surgery categories (*P*<0.05 for each category), ranging from 0.08% for PTCA to 0.74% for heart valve procedures per year. Particularly after adjustment, annualized quality growth rate of tracheostomy changed to significant, and the quality growth rate of PTCA became significantly positive. After adjusting cost for the same factors used to adjust quality, surgeries with a significant decrease in cost include tracheostomy, heart valve procedures, small bowel resection, colorectal resection, excision/lysis peritoneal adhesions, and debridement of wound (*P*<0.05 for each category, Fig 2B). Conversely, costs increased significantly for CABG, PTCA, and gastrostomy (*P*<0.05) even after adjustment. Changes in cost range from -2.59% (tracheostomy) to 1.11% (PTCA) per year.

Adjusted regression data further reveal that tracheostomy, heart valve procedures, small bowel resection, and debridement of wound simultaneously displayed a significant increase in quality and a significant decrease in cost from 2002–2015. In contrast, colorectal resection and excision/lysis peritoneal adhesions showed a significant decrease in cost with no significant change in quality. Ileostomy/other enterostomy and exploratory laparotomy had no significant changes in both quality and cost over 2002–2015. Adjusted quality and cost both increased significantly for CABG, PTCA, and gastrostomy (Fig 2C).

Favorable trends in overall quality (survival without readmission) were driven mainly by improvements in survival in some cases (tracheostomy, gastrostomy, and small bowel resection) and in others by lower rates of unplanned readmissions (heart valve procedures and CABG), as shown in S3 Fig. Both aspects of quality contributed for wound debridement, while reductions in mortality were offset by increases in readmissions for colorectal resection and excision of lysis peritoneal adhesions.

The interaction between patient age and the indicator variable for the year 2015 was significant (at a 10% level) for 4 of the 11 analyses of surgical costs and 3 of the 11 analyses of surgical quality (S4 Fig). In all 4 of these cost analyses, costs declined more rapidly for older individuals. For example, costs decreased by an annual rate of 0.81% for 80-year-olds undergoing heart valve procedures, versus 0.48% for 70-year-olds. The results for quality trends did not show such a clear pattern.

**Fig 3:**
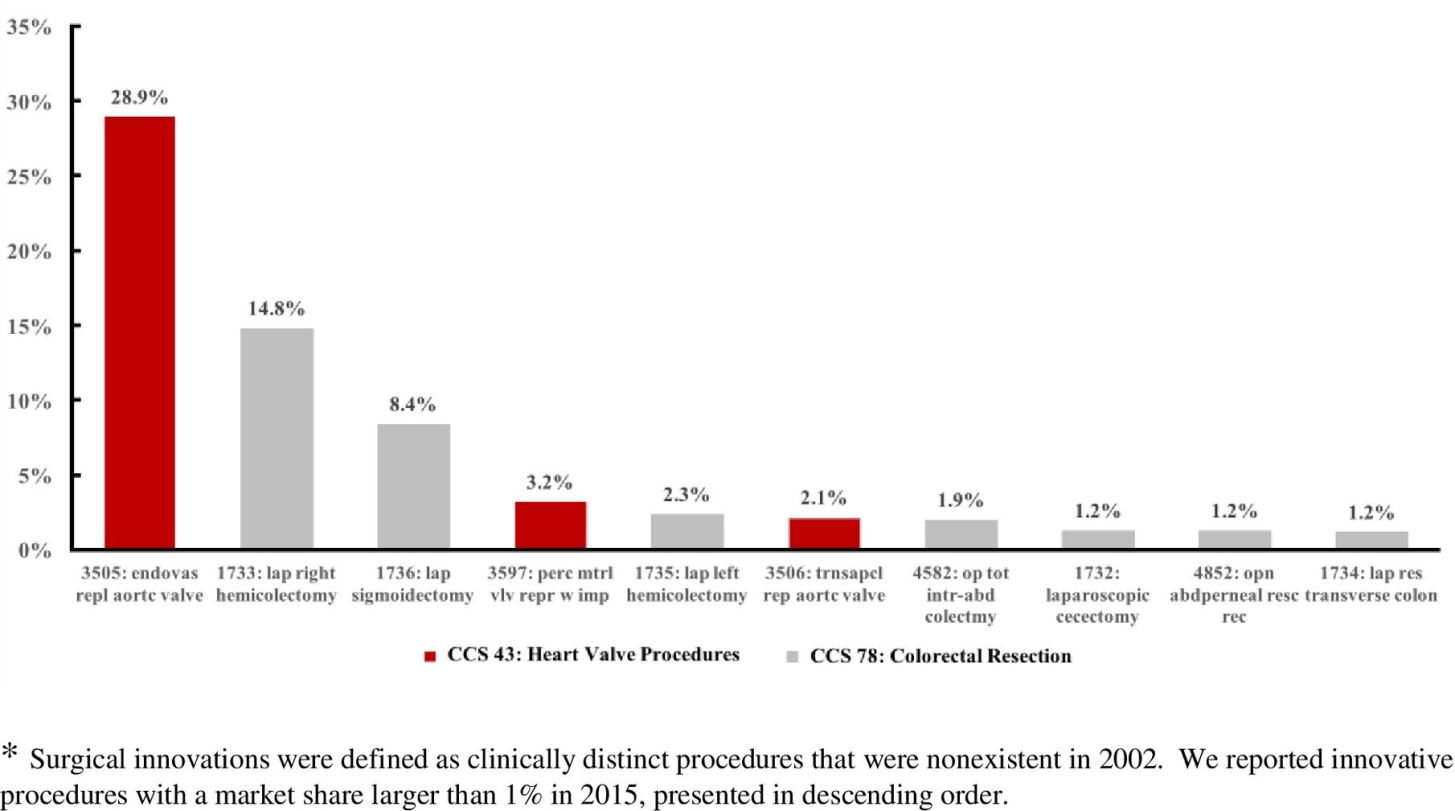
Top shares of innovative ICD-9-CM procedures in 2015, compared with 2002.

**Fig 4:**
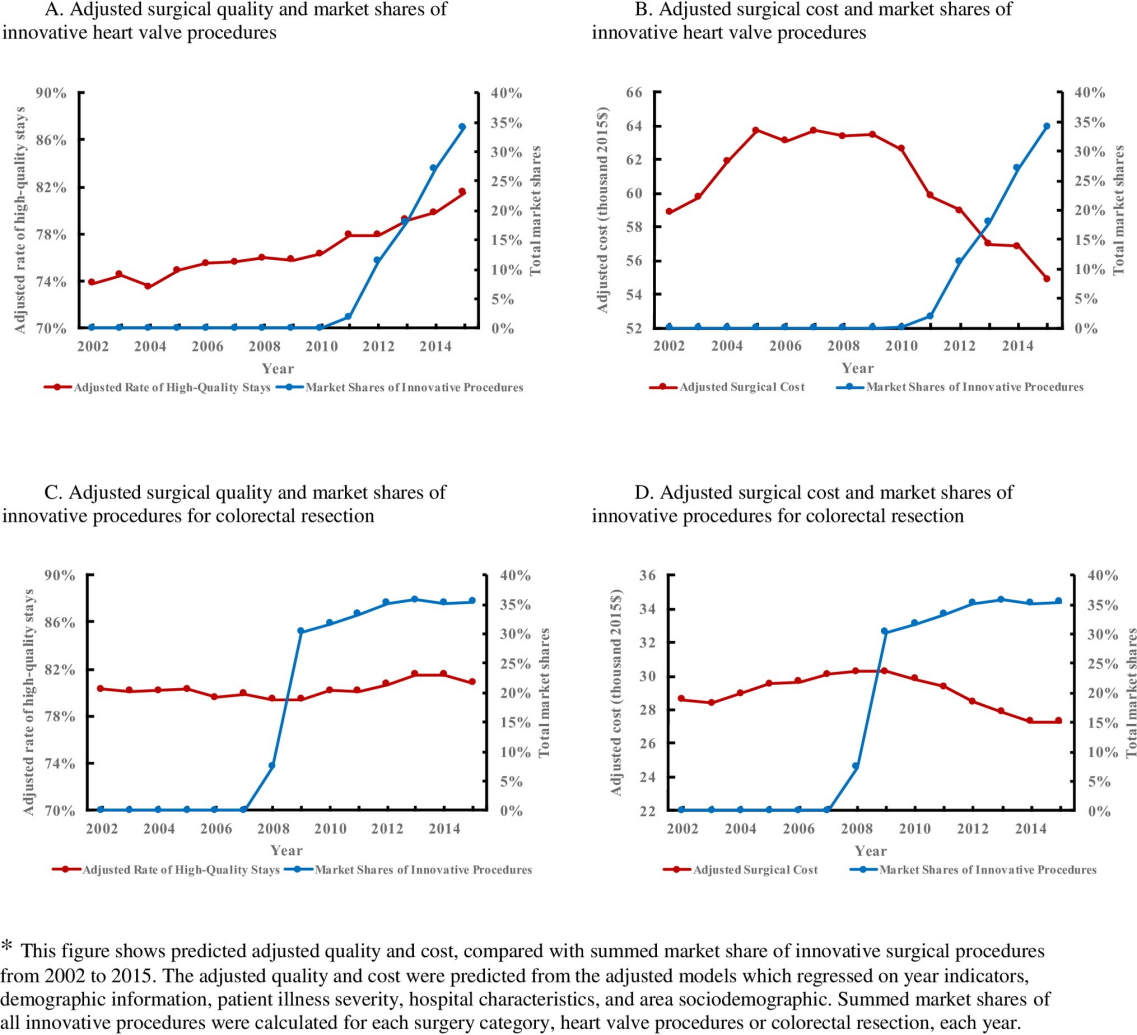
Adjusted surgical quality, adjusted cost, and market shares of innovative procedures, 2002–2015. (A) Adjusted surgical quality and market shares of innovative heart valve procedures. (B) Adjusted surgical cost and market shares of innovative heart valve procedures. (C) Adjusted surgical quality and market shares of innovative procedures for colorectal resection. (D) Adjusted surgical cost and market shares of innovative procedures for colorectal resection.

The decrease in cost of certain surgeries may be associated with the use of innovative surgical procedures. Among the 11 surgery categories, only 3 surgery categories had new procedure codes which were not used in 2002 (S2 Table). What is more, the new procedure codes for PTCA were converted from pervious ICD-9 codes, so that only heart valve procedures and colorectal resection showed increased use of novel procedures from 2002–2015. Novel procedures with the market shares greater than 1% in 2015 were presented in Fig 3. For heart valve procedures, endovascular (ICD-9-CM 3505), percutaneous mitral valve repair with implant (ICD-9-CM 3597), and transapical (ICD-9-CM 3506) aortic valve replacements increased in frequency to 28.9%, 3.2%, and 2.1%, respectively, by 2015. For colorectal resection, frequencies of laparoscopic right hemicolectomy (ICD-9-CM 1733), laparoscopic sigmoidectomy (ICD-9-CM 1736), and laparoscopic left hemicolectomy (ICD-9-CM 1735) reached frequencies of 14.8%, 8.4%, and 2.3%, respectively, by 2015. By comparing the adjusted surgical quality and cost to total shares of all corresponding novel procedures, we found that slight increases in surgical quality and substantial decreases in costs occurred around the years when novel procedures were introduced to the market (Fig 4).

Similar results were obtained from sensitivity analyses in which we required 30-day survival after discharge for defining high-quality stays. Adjusted redefined quality increased for all CCS surgery categories, the significant improvement was shown for the same 7 surgical categories. Adjusted growth rates of quality increased for all surgical categories. Unadjusted and adjusted growth rates of daily hospitalization cost were positive for all CCS surgery categories except tracheostomy (S5 Fig), indicating that the length of hospital stays is one of the main factors that drove down total surgical cost per stay. For patients with heart attack, controlling for the location of the heart attack and the likelihood of inpatient mortality revealed trends in quality over the study period that are consistent with our initial results (S6 Fig). Our findings were also similar when we excluded the small proportion (0.35%) of all surgeries that were apparently mis-coded as newborn admissions (S7 Fig).

## Discussion

In this study, we assessed trends in cost and quality for 11 surgery categories involving inpatient hospital stays from 2002–2015. Our unadjusted data reveal general improvement in quality for all categories, except for PTCA, colorectal resection, excision/lysis peritoneal adhesions, ileostomy and other enterostomy. In contrast, unadjusted costs of CABG, PTCA, gastrostomy, and exploratory laparotomy increased, whereas those of the other seven categories decreased over the study period. Notably, adjustment for health-related covariates resulted in higher quality metrics for 8 surgery categories. Adjustment for disease severity and patient outcomes further revealed significant trends in quality for tracheostomy, gastrostomy, small bowel resection, and PTCA that were not apparent in the unadjusted data. In particular, growth in quality for PTCA changed from significantly negative to significantly positive after adjustment. Adjustment also impacted cost trends to some extent. Specifically, compared with unadjusted cost metrics, adjusted metrics were lower for all surgery categories except tracheostomy and heart valve procedures. These results indicate that health-related covariates are important drivers of changes in surgical quality and cost, with the potential to mask real changes in these metrics. A possible explanation is that patient demographics changed over the 14-year study period. Physician skill and technologies may also have improved, allowing more seriously ill patients to undergo certain surgeries. Furthermore, patients with less severe conditions have been increasingly moved to outpatient sectors. Therefore, in the later years of the study, those with inpatient stays might be, on average, more unwell than those at the beginning of the study. These changes may have led to worse apparent quality outcomes and higher surgical costs per stay.

Adjusting the data for illness severity resulted in consistent improvement in surgical quality over the study period for all 11 categories. This may have resulted from changes in physician experience, improved quality assurance, and technological innovations [6]. We further speculate that surgeons could have gained experience in preforming surgeries and making decisions based on patients’ conditions over the 14-year study period. Improved quality assurances, such as improved antibiotic timing and glucose control, prevention of hypothermia, and reduction of unnecessary transfusions, would help to control complications, readmissions, and deaths at early stages [6]. After adjusting for illness severity, surgical costs declined for nine categories, and significantly increased only for CABG and PTCA. Here, we speculate that innovations may have effectively controlled costs and improved quality for certain surgeries. Notably, however, among the 11 surgery categories, novel procedures came into use during the study period only for heart valve procedures and colorectal resections. For heart valve procedures, there was a relative increase in use of aortic valve surgery *vs*. mitral valve surgery and an increased use of less invasive procedures. For colorectal resection, there was a major shift from open procedures to laparoscopic procedures. These newer and less invasive procedures require only small incisions, which minimizes the burden on both patients and hospitals, leading to quicker recoveries and lower costs.

Our data further reveal that adjusted costs increased significantly for two surgery categories, CABG and PTCA. Although quality improved significantly for both of these categories over the study period, costs increased at a faster rate than quality. Thus, it is difficult to ascertain whether the increased cost was worth the improvements in quality. The use of productivity metrics to further assess changes in the values of these surgeries might help to determine if the changes implemented over the study period were truly beneficial.

Our findings are similar to those of previous studies, including Weiser *et al*. (2011) and Romley *et al*. (2015), which reported quality improvements over time and provided more up-to-date information on both surgical cost and quality relative to the previous literature. However, our analysis has some limitations. First, our quality metrics did not include quality of life, functional status, life expectancy, complications, time to return to the community, or clinical biomarkers, all of which can be used to quantify quality of care. For example, one previous study used patient return to the community to evaluate productivity of skilled nursing facilities [23]. Nevertheless, survival without unplanned readmission is a meaningful quality metric that has received increasing attention from patients, physicians, and policy makers [19]. A second limitation is that our sample size decreased from 2002–2015. One possible explanation is that surgeons might have become less inclined to perform certain surgeries. Additionally, some surgeries have moved from the inpatient to the outpatient sector and therefore, were no longer captured by our data. Increasing utilization of outpatient services means that patients with less severe conditions are not admitted to hospitals. Because outpatient care is generally associated with lower costs and lower rates of readmission and mortality, this could lead us to underestimate reduction in cost and increase in quality of surgical care. Nevertheless, it would be informative to perform sensitivity analyses including surgical services from outpatient sectors in future studies. A third limitation is that the trends documented through September, 2015 may not generalize to the period after, when a new coding scheme (ICD-10-CM) became mandatory. While the validity of comparisons before and after this coding transition would be uncertain, analyzing trends from late 2015 onward represents a worthwhile direction for further study. Lastly, although we descriptively characterized innovations from 2002–2015, we did not explore causal relationships among quality, cost, and innovations, which could be informative in future research.

## Conclusions

Our results suggest significant quality improvement for 7 surgery categories over the study period. Costs decreased significantly for 6 of the 11 categories, although they increased significantly for 3 categories. Future work can further explore the value of inpatient surgeries, particularly when surgical cost and quality increase simultaneously.

## Supporting information

S1 Table Demographic information for hospitalized patients who underwent a surgical intervention, 2002–2015. (DOCX)

S2 Table Top shares of new ICD-9-CM procedures in 2015, compared with 2002. (DOCX)

S3 Table Regression results for cost and quality of CCS 34 tracheostomy on a year indicator. (A) Regression results for cost of CCS 34 tracheostomy on a year indicator. (B) Regression results for quality of CCS 34 tracheostomy on a year indicator.
(DOCX)

S4 Table Regression results for cost and quality of CCS 43 heart valve procedures on a year indicator. (A) Regression results for cost of CCS 43 heart valve procedures on a year indicator. (B) Regression results for quality of CCS 43 heart valve procedures on a year indicator.
(DOCX)

S5 Table Regression results for cost and quality of CCS 44 coronary artery bypass grafting on a year indicator. (A) Regression results for cost of CCS 44 coronary artery bypass grafting on a year indicator. (B) Regression results for quality of CCS 44 coronary artery bypass grafting on a year indicator.
(DOCX)

S6 Table Regression results for cost and quality of CCS 45 percutaneous transluminal coronary angioplasty on a year indicator. (A) Regression results for cost of CCS 45 percutaneous transluminal coronary angioplasty on a year indicator. (B) Regression results for quality of CCS 45 percutaneous transluminal coronary angioplasty on a year indicator.
(DOCX)

S7 Table Regression results for cost and quality of CCS 71 gastrostomy on a year indicator. (A) Regression results for cost of CCS 71 gastrostomy on a year indicator. (B) Regression results for quality of CCS 71 gastrostomy on a year indicator.
(DOCX)

S8 Table Regression results for cost and quality of CCS 73 ileostomy and other enterostomy on a year indicator. (A) Regression results for cost of CCS 73 ileostomy and other enterostomy on a year indicator. (B) Regression results for quality of CCS 73 ileostomy and other enterostomy on a year indicator.
(DOCX)

S9 Table Regression results for cost and quality of CCS 75 small bowel resection on a year indicator. (A) Regression results for cost of CCS 75 small bowel resection on a year indicator. (B) Regression results for quality of CCS 75 small bowel resection on a year indicator.
(DOCX)

S10 Table Regression results for cost and quality of CCS 78 colorectal resection on a year indicator. (A) Regression results for cost of CCS 78 colorectal resection on a year indicator. (B) Regression results for quality of CCS 78 colorectal resection on a year indicator.
(DOCX)

S11 Table Regression results for cost and quality of CCS 89 exploratory laparotomy on a year indicator. (A) Regression results for cost of CCS 89 exploratory laparotomy on a year indicator. (B) Regression results for quality of CCS 89 exploratory laparotomy on a year indicator.
(DOCX)

S12 Table Regression results for cost and quality of CCS 90 excision, lysis peritoneal adhesions on a year indicator. (A) Regression results for cost of CCS 90 excision, lysis peritoneal adhesions on a year indicator. (B) Regression results for quality of CCS 90 excision, lysis peritoneal adhesions on a year indicator.
(DOCX)

S13 Table Regression results for cost and quality of CCS 169 debridement of wound on a year indicator. (A) Regression results for cost of CCS169 debridement of wound on a year indicator. (B) Regression results for quality of CCS169 debridement of wound on a year indicator.
(DOCX)

S1 Fig Trends of unadjusted and adjusted quality for each surgical category, 2002–2015. (PDF)

S2 Fig Trends of unadjusted and adjusted cost for each surgical category, 2002–2015. (PDF)

S3 Fig Decomposition of overall quality trends into survival and avoidance of unplanned readmissions. (PDF)

S4 Fig Sensitivity analysis: Interacting year indicator with patient age. (PDF)

S5 Fig Sensitivity analysis: Unadjusted and adjusted annualized growth rate of quality and cost with redefined quality and cost metrics. (A) Unadjusted and adjusted annualized growth rate of surgical quality in sensitivity analysis (quality redefined as rate of surviving 30+ days after discharge without unplanned readmission). (B) Unadjusted and adjusted annualized growth rate of surgical cost (cost redefined as cost per day in hospital). (C) Unadjusted and adjusted annualized change rate of days in hospitals.
(PDF)

S6 Fig Sensitivity analysis: Adjusted annualized growth rate of quality and cost before and after adjusting for extra risk factors for patients with a heart attack during hospitalizations. (PDF)

S7 Fig Sensitivity analysis: Excluding newborn admissions. (PDF)
