# Supplementary material for: Trends in the quality and cost of inpatient surgical procedures in the United States, 2002–2015
Source: PLoS One. 2021 Nov 3;16(11):e0259011. doi: 10.1371/journal.pone.0259011 (PMC8565758; doi:10.1371/journal.pone.0259011)
Supplement: S1 Table — (DOCX) [file pone.0259011.s001.docx]

**S1 Table.** Demographic Information for Hospitalized Patients Who Underwent a Surgical Intervention, 2002-2015

|  | **CCS 34** | **CCS 43** | | **CCS 44** | | **CCS 45** | | **CCS 71** | **CCS 73** | **CCS 75** | | **CCS 78** | | **CCS 89** | | **CCS 90** | **CCS169** |  |  |
| --- | --- | --- | --- | --- | --- | --- | --- | --- | --- | --- | --- | --- | --- | --- | --- | --- | --- | --- | --- |
| Variable | Tracheostomy | Heart Valve Procedures | | CABG | | PTCA | | Gastrostomy | Ileostomy and Other Enterostomy | Small Bowel Resection | | Colorectal Resection | | Exploratory Laparotomy | | Excision, Lysis Peritoneal Adhesions | Debridement of Wound |  |  |
| Inpatient stay case number, n | 46,689 | 111,333 | | 196,061 | | 526,351 | | 100,026 | 5,418 | 46,067 | | 231,575 | | 7,413 | | 51,373 | 110,206 |  |  |
| Hospital number, n | 2,716 | 1,277 | | 1,303 | | 1,909 | | 3,405 | 1,814 | 3,138 | | 3,546 | | 2,164 | | 3,215 | 3,581 |  |  |
| Rate of high-quality surgical stays (%) ª | 69.7% | 76.6% | | 81.9% | | 87.6% | | 57.7% | 54.3% | 72.7% | | 80.2% | | 52.3% | | 81.3% | 74.5% |  |  |
| Inpatient costs, 2015 $ | 85210.5 | 52995.8 | | 40592.5 | | 19150.1 | | 17408.1 | 24631.1 | 32272.8 | | 24949.2 | | 25066.0 | | 23305.3 | 15835.9 |  |  |
| Part B non-institutional costs, 2015 $ | 10680.7 | 7587.4 | | 6401.9 | | 2035.5 | | 2780.0 | 3608.6 | 4656.0 | | 4178.7 | | 3647.8 | | 3343.2 | 2421.9 |  |  |
| Total costs, 2015 $ | 95906.0 | 60595.3 | | 46999.0 | | 21196.3 | | 20176.7 | 28331.1 | 36937.9 | | 29131.3 | | 28776.5 | | 26722.8 | 18250.4 |  |  |
| Length of stay, day | 30.4 | 11.9 | | 10.7 | | 4.2 | | 12.2 | 13.2 | 14.0 | | 11.3 | | 10.3 | | 11.6 | 10.6 |  |  |
| Teaching hospital (%) ᵇ | 77.4% | 83.5% | | 69.4% | | 67.7% | | 55.3% | 64.7% | 56.5% | | 54.4% | | 65.0% | | 52.8% | 55.7% |  |  |
| **Patient Characteristics** |  |  | |  | |  | |  |  |  | |  | |  | |  |  |  |  |
| Age, mean (SD) | 76.1 (7.2) | 76.8 (6.7) | | 74.0 (5.8) | | 75.4 (6.7) | | 81.7 (8.0) | 77.3 (7.8) | 77.7 (7.7) | | 76.8 (7.3) | | 76.7 (7.4) | | 76.9 (7.6) | 78.0 (8.2) |  |  |
| Female (%) | 48.4% | 44.8% | | 32.1% | | 42.0% | | 55.6% | 45.6% | 61.7% | | 58.4% | | 56.6% | | 64.2% | 57.3% |  |  |
| Race: African American (%) | 17.3% | 3.7% | | 5.1% | | 5.8% | | 21.5% | 12.1% | 8.8% | | 7.8% | | 8.9% | | 9.4% | 15.4% |  |  |
| Race: Hispanic (%) | 2.6% | 1.1% | | 1.4% | | 1.4% | | 3.5% | 2.2% | 1.5% | | 1.2% | | 1.8% | | 1.5% | 2.5% |  |  |
| Race: White (%) | 75.7% | 93.1% | | 90.6% | | 90.1% | | 70.8% | 82.1% | 87.3% | | 88.7% | | 86.4% | | 87.1% | 79.7% |  |  |
| Race: other (%) | 4.3% | 2.1% | | 2.9% | | 2.6% | | 4.2% | 3.6% | 2.4% | | 2.4% | | 3.0% | | 2.0% | 2.4% |  |  |
| **Social Characteristics ᵈ** |  |  | |  | |  | |  |  |  | |  | |  | |  |  |  |  |
| Median household income, mean (SD) | 41,940 (16,224) | 45,950 (17,554) | | 43,008  (15,793) | | 43,409 (16,015) | | 41,861 (16,456) | 43,609 (16,276) | 44,222 (16,594) | | 44,342 (16,651) | | 42,092 (15,407) | | 44,350 (16,757) | 42,140 (16,091) |  |  |
| Social Security income, mean (SD) | 11,121 (1,576) | 11,610 (1,486) | | 11,351  (1,469) | | 11,393 (1,466) | | 11,052 (1,584) | 11,300 (1,497) | 11,414 (1,500) | | 11,442 (1,486) | | 11,242 (1,498) | | 11,419 (1,479) | 11,171 (1,550) |  |  |
| Poor (%) | 13.8% | 10.6% | | 11.6% | | 11.5% | | 14.0% | 12.2% | 11.5% | | 11.3% | | 12.4% | | 11.5% | 13.2% |  |  |
| Employed (%) | 93.4% | 94.7% | | 94.5% | | 94.5% | | 93.4% | 94.0% | 94.4% | | 94.5% | | 94.1% | | 94.5% | 93.7% |  |  |
| Less than high school education (%) | 21.9% | 17.9% | | 19.6% | | 19.4% | | 22.2% | 19.7% | 18.9% | | 18.8% | | 20.3% | | 18.9% | 21.2% |  |  |
| Urban (%) | 77.2% | 71.9% | | 68.4% | | 70.1% | | 78.0% | 75.6% | 73.2% | | 72.6% | | 70.4% | | 73.2% | 74.3% |  |  |
| Hispanic (%) | 11.6% | 8.0% | | 8.2% | | 8.2% | | 12.4% | 10.1% | 8.8% | | 8.3% | | 9.0% | | 8.7% | 10.6% |  |  |
| Single (%) | 44.0% | 40.8% | | 40.7% | | 41.1% | | 44.0% | 42.6% | 41.7% | | 41.5% | | 42.0% | | 41.7% | 43.2% |  |  |
| Elderly in an institution (%) | 5.5% | 5.4% | | 5.5% | | 5.5% | | 5.9% | 5.7% | 5.6% | | 5.6% | | 5.5% | | 5.6% | 5.7% |  |  |
| Non-institutionalized elderly with physical disability (%) | 30.1% | 27.9% | | 29.2% | | 29.1% | | 30.4% | 29.2% | 28.8% | | 28.7% | | 29.4% | | 28.8% | 29.9% |  |  |
| Sensory disability among elderly (%) | 14.6% | 14.0% | | 14.6% | | 14.5% | | 14.6% | 14.4% | 14.3% | | 14.3% | | 14.7% | | 14.3% | 14.6% |  |  |
| Mental disability (%) | 11.7% | 10.2% | | 10.9% | | 10.8% | | 12.0% | 11.1% | 10.7% | | 10.7% | | 11.1% | | 10.7% | 11.4% |  |  |
| Self-care disability (%) | 10.4% | 9.1% | | 9.6% | | 9.5% | | 10.6% | 9.7% | 9.5% | | 9.4% | | 9.8% | | 9.5% | 10.2% |  |  |
| Difficulty going-outside-the-home disability (%) | 21.7% | 19.4% | | 20.2% | | 20.2% | | 22.0% | 20.7% | 20.1% | | 20.0% | | 20.6% | | 20.1% | 21.3% |  |  |
| **Patient Disease Severity** |  |  | |  | |  | |  |  |  | |  | |  | |  |  |  |  |
| Number of Charlson-Deyo comorbidities, mean (SD) | 1.4 (1.0) | 1.1 (0.9) | | 1.3 (1.0) | | 1.3 (1.0) | | 1.4 (1.0) | 1.3 (1.0) | 0.9 (0.9) | | 1.3 (1.1) | | 1.3 (1.0) | | 0.8 (0.9) | 1.2 (1.0) |  |  |
| 1 Charlson-Deyo comorbidity (%) | 36.8% | 40.0% | | 37.4% | | 37.1% | | 33.8% | 36.5% | 35.4% | | 34.7% | | 35.1% | | 35.3% | 37.1% |  |  |
| 2 Charlson-Deyo comorbidities (%) | 29.2% | 23.2% | | 26.1% | | 25.2% | | 28.0% | 28.3% | 17.1% | | 26.0% | | 26.1% | | 15.3% | 24.2% |  |  |
| 3 Charlson-Deyo comorbidities (%) | 10.7% | 7.0% | | 10.3% | | 10.8% | | 12.3% | 9.7% | 4.2% | | 10.0% | | 9.0% | | 3.7% | 8.8% |  |  |
| 4 Charlson-Deyo comorbidities (%) | 1.9% | 1.1% | | 2.2% | | 2.8% | | 3.0% | 2.0% | 0.6% | | 2.3% | | 2.4% | | 0.6% | 1.7% |  |  |
| 5+ Charlson-Deyo comorbidities (%) | 0.3% | 0.1% | | 0.3% | | 0.4% | | 0.4% | 0.1% | 0.1% | | 0.3% | | 0.3% | | 0.0% | 0.2% |  |  |
| Transferred from other hospital (%) | 9.7% | 8.7% | | 15.6% | | 14.3% | | 3.7% | 4.3% | 4.4% | | 1.9% | | 5.9% | | 3.4% | 2.6% |  |  |
| Emergency inpatient admissions (%) | 64.5% | 14.1% | | 24.7% | | 39.3% | | 70.5% | 48.2% | 60.2% | | 28.6% | | 52.4% | | 55.3% | 53.7% |  |  |
| Urgent inpatient admissions (%) | 18.0% | 17.2% | | 26.1% | | 27.7% | | 19.4% | 21.0% | 18.6% | | 14.8% | | 18.2% | | 18.4% | 25.3% |  |  |
| Elective inpatient admissions (%) | 16.0% | 68.5% | | 48.9% | | 32.6% | | 9.7% | 30.4% | 20.9% | | 56.4% | | 28.3% | | 26.1% | 20.5% |  |  |
| **Heart Attack Related Risk Factor Adjustors (CCS 44) ᵍ** |  | |  | |  | |  | |  | |  | |  | |  | |  | |  |
| Cases with AHRQ predicted inpatient mortality, n (%) | - | - | | 43,537 | | - | | - | - | - | | - | | - | | - | - |  |  |
| AHRQ predicted inpatient mortality (%) | - | - | | 2.7% | | - | | - | - | - | | - | | - | | - | - |  |  |
| Location of heart attack: ICD-9 410.0x Anterolateral, n (%) | - | - | | 1,977 (4.5%) | | - | | - | - | - | | - | | - | | - | - |  |  |
| Location of heart attack: ICD-9 410.1x Other Anterior Wall, n (%) | - | - | | 3,398 (7.8%) | | - | | - | - | - | | - | | - | | - | - |  |  |
| Location of heart attack: ICD-9 410.2x Inferolateral Wall, n(%) | - | - | | 851 (2.0%) | | - | | - | - | - | | - | | - | | - | - |  |  |
| Location of heart attack: ICD-9 410.3x Inferoposterior Wall, n(%) | - | - | | 565 (1.3%) | | - | | - | - | - | | - | | - | | - | - |  |  |
| Location of heart attack: ICD-9 410.4x Other Inferior Wall, n(%) | - | - | | 4,289 (9.9%) | | - | | - | - | - | | - | | - | | - | - |  |  |
| Location of heart attack: ICD-9 410.5x Other Lateral Wall, n(%) | - | - | | 451 (1.0%) | | - | | - | - | - | | - | | - | | - | - |  |  |
| Location of heart attack: ICD-9 410.6x True Posterior Wall, n(%) | - | - | | 170 (0.4%) | | - | | - | - | - | | - | | - | | - | - |  |  |
| Location of heart attack: ICD-9 410.7x Sub-Endocardial, n(%) | - | - | | 29,443 (67.6%) | | - | | - | - | - | | - | | - | | - | - |  |  |
| Location of heart attack: ICD-9 410.8x Other Specified Sites, n(%) | - | - | | 408 (0.9%) | | - | | - | - | - | | - | | - | | - | - |  |  |
| Location of heart attack: ICD-9 410.9x Unspecified site, n(%) | - | - | | 1,983 (4.6%) | | - | | - | - | - | | - | | - | | - | - |  |  |

Abbreviations: CCS, Clinical Classifications System; CABG, coronary artery bypass grafting; PTCA, percutaneous transluminal coronary angioplasty; AHRQ, the Agency for Healthcare Research and Quality

ª A high-quality surgical stay was defined a stay in which a patient survived for at least 30 days after his/her initial hospital admission and was not readmitted with an unplanned readmission within 30 days of discharge.

^b^ Teaching hospitals were defined as those with a resident-to-bed ratio larger than 0.6.

^d^ Community-level social characteristics were integrated from the 2010 US Census data.

^e^ Together with patients without Charlson-Deyo comorbidity, the numbers summed up to 100%.

^g^ Heart attack related risks were applied to coronary artery bypass grafting only (CCS code 44, CABG), n= 195,441.
