# Supplementary material for: Trends in the quality and cost of inpatient surgical procedures in the United States, 2002–2015
Source: PLoS One. 2021 Nov 3;16(11):e0259011. doi: 10.1371/journal.pone.0259011 (PMC8565758; doi:10.1371/journal.pone.0259011)
Supplement: S2 Table — (DOCX) [file pone.0259011.s002.docx]

**S6 Table.** Top Shares of New ICD-9-CM Procedures in 2015, Compared with 2002*

| **Surgery** | **ICD-9-CM procedure code** | **Market share in 2015** | **Summed market share**  **in 2015** |
| --- | --- | --- | --- |
| CCS43 Heart Valve Procedures | 3505 | 28.9% | 34.2% |
|  | 3506 | 2.1% |  |
|  | 3597 | 3.2% |  |
| CCS45 Percutaneous Transluminal Coronary Angioplasty | 00.66 | 99.8% | 100% |
|  | 17.55 | 0.2% |  |
| CCS78 Colorectal Resection | 1732 | 1.2% | 33.0% |
|  | 1733 | 14.8% |  |
|  | 1734 | 1.2% |  |
|  | 1735 | 2.3% |  |
|  | 1736 | 8.4% |  |
|  | 1739 | 1.0% |  |
|  | 4582 | 1.9% |  |
|  | 4851 | 1.0% |  |
|  | 4852 | 1.2% |  |

* These procedures were nonexistent in 2002. The 5 most commonly used procedures for each CCS surgery category were listed in the table. Among them, ICD-9 00.66 for PTCA was converted from ICD-9 36.01, 36.02, and 36.05 in 2005, and was converted to ICD-9 17.55 in 2011. So that ICD-9 0.66 and 17.55 are not innovative procedures by definition.
