# Supplementary material for: Trends in the quality and cost of inpatient surgical procedures in the United States, 2002–2015
Source: PLoS One. 2021 Nov 3;16(11):e0259011. doi: 10.1371/journal.pone.0259011 (PMC8565758; doi:10.1371/journal.pone.0259011)
Supplement: S3 Table — (A) Regression results for cost of CCS 34 tracheostomy on a year indicator. (B) Regression results for quality of CCS 34 tracheostomy on a year indicator. (DOCX) [file pone.0259011.s003.docx]

**S10 Table.** Regression Results for Cost and Quality of CCS 34 Tracheostomy on a Year Indicator

S10A Table. Regression results for cost of CCS 34 tracheostomy on a year indicator

| Cost of CCS 34 | Coefficient | Robust standard error | P-value | 95% confidence interval |
| --- | --- | --- | --- | --- |
| Year 2015 | -40.77 | 2.34 | < 0.001 | (-45.35, -36.19) |
| Age | -0.43 | 0.08 | < 0.001 | (-0.59, -0.26) |
| Race (Ref = White) |  |  |  |  |
| Black | -2.38 | 2.21 | 0.282 | (-6.71, 1.96) |
| Asian | 12.02 | 5.31 | 0.024 | (1.60, 22.43) |
| Hispanic | 2.70 | 4.66 | 0.563 | (-6.45, 11.84) |
| Female | -3.44 | 1.15 | 0.003 | (-5.69, -1.19) |
| Number of Charlson-Deyo comorbidity (Ref = 0) |  |  |  |  |
| 1 | -2.69 | 1.81 | 0.138 | (-6.25, 0.87) |
| 2 | -5.60 | 1.98 | 0.005 | (-9.49, -1.72) |
| 3 | -10.13 | 2.34 | < 0.001 | (-14.73, -5.54) |
| 4 | -7.59 | 4.98 | 0.128 | (-17.36, 2.19) |
| 5 | -12.46 | 11.62 | 0.284 | (-35.26, 10.33) |
| Teaching hospital | 5.10 | 1.85 | 0.006 | (1.47, 8.73) |
| Transferred from other hospitals | 4.57 | 2.59 | 0.077 | (-0.50, 9.64) |
| Transferred to other hospitals | -5.96 | 1.90 | 0.002 | (-9.69, -2.23) |
| Social Characteristics |  |  |  |  |
| % urban in the community | -5.41 | 3.04 | 0.075 | (-11.37, 0.54) |
| % of the employed in the community | 44.27 | 26.74 | 0.098 | (-8.18, 96.72) |
| % Hispanic in the community | 21.95 | 8.01 | 0.006 | (6.24, 37.66) |
| % single in the community | 20.07 | 16.36 | 0.220 | (-12.00, 52.15) |
| % of the poor in the community | 32.14 | 18.27 | 0.079 | (-3.69, 67.97) |
| Social Security income | 0.30 | 0.60 | 0.614 | (-0.87, 1.48) |
| Median household income | 0.29 | 0.09 | 0.002 | (0.11, 0.46) |
| % with education less than high school | -20.36 | 14.07 | 0.148 | (-47.97, 7.24) |
| % sensory disability among elderly | -9.12 | 17.15 | 0.595 | (-42.77, 24.52) |
| % non-institutionalized elderly with physical disability | -14.79 | 14.67 | 0.313 | (-43.57, 13.98) |
| % people with mental disability in the community | 63.70 | 22.64 | 0.005 | (19.29, 108.11) |
| % people with self-care disability | -7.64 | 25.24 | 0.762 | (-57.14, 41.86) |
| % people with difficulty going-outside-the-home disability | -18.09 | 18.66 | 0.332 | (-54.68, 18.51) |
| % elderly in an institution | 2.62 | 11.26 | 0.816 | (-19.46, 24.70) |
| Admission type (Ref = Emergency) |  |  |  |  |
| Urgent | 6.02 | 2.15 | 0.005 | (1.80, 10.24) |
| Elective | 5.19 | 2.23 | 0.020 | (0.82, 9.56) |
| Newborn | 0.90 | 6.18 | 0.884 | (-11.21, 13.01) |
| Diagnosis codes | Included | Included | Included | Included |
| Constant | 94.67 | 31.23 | 0.002 | (33.43, 155.91) |
|  |  |  |  |  |
| Number of observations: 7,159  R-squared: 0.19  Root MSE: 48.75 | | | | |

S10B Table. Regression results for quality of CCS 34 tracheostomy on a year indicator

| Quality of CCS 34 | Coefficient | Robust standard error | P-value | 95% confidence interval |
| --- | --- | --- | --- | --- |
| Year 2015 | 0.19 | 0.08 | 0.013 | (0.04, 0.34) |
| Age | -0.01 | 0.00 | 0.170 | (-0.01, 0.00) |
| Race (Ref = White) |  |  |  |  |
| Black | 0.05 | 0.08 | 0.566 | (-0.12, 0.21) |
| Asian | 0.19 | 0.14 | 0.178 | (-0.09, 0.47) |
| Hispanic | 0.14 | 0.19 | 0.461 | (-0.24, 0.52) |
| Female | 0.09 | 0.05 | 0.108 | (-0.02, 0.19) |
| Number of Charlson-Deyo comorbidity (Ref = 0) |  |  |  |  |
| 1 | -0.02 | 0.08 | 0.777 | (-0.17, 0.13) |
| 2 | -0.07 | 0.08 | 0.387 | (-0.23, 0.09) |
| 3 | -0.16 | 0.11 | 0.121 | (-0.37, 0.04) |
| 4 | -0.20 | 0.19 | 0.307 | (-0.58, 0.18) |
| 5 | 0.04 | 0.52 | 0.940 | (-0.99, 1.06) |
| Teaching hospital | 0.10 | 0.05 | 0.042 | (0.00, 0.19) |
| Transferred from other hospitals | 0.06 | 0.09 | 0.476 | (-0.11, 0.24) |
| Transferred to other hospitals | 0.85 | 0.09 | < 0.001 | (0.68, 1.02) |
| Social Characteristics |  |  |  |  |
| % urban in the community | -0.12 | 0.11 | 0.280 | (-0.34, 0.10) |
| % of the employed in the community | 0.10 | 1.10 | 0.931 | (-2.06, 2.25) |
| % Hispanic in the community | 0.15 | 0.22 | 0.471 | (-0.27, 0.58) |
| % single in the community | 0.53 | 0.49 | 0.283 | (-0.44, 1.50) |
| % of the poor in the community | -0.36 | 0.68 | 0.598 | (-1.68, 0.97) |
| Social Security income | -0.01 | 0.02 | 0.555 | (-0.06, 0.03) |
| Median household income | 0.00 | 0.00 | 0.459 | (0.00, 0.01) |
| % with education less than high school | -0.47 | 0.48 | 0.336 | (-1.41, 0.48) |
| % sensory disability among elderly | 0.50 | 0.89 | 0.576 | (-1.24, 2.23) |
| % non-institutionalized elderly with physical disability | -0.03 | 0.67 | 0.962 | (-1.34, 1.27) |
| % people with mental disability in the community | -0.02 | 0.96 | 0.981 | (-1.90, 1.85) |
| % people with self-care disability | 1.32 | 1.20 | 0.273 | (-1.04, 3.68) |
| % people with difficulty going-outside-the-home disability | -0.77 | 0.85 | 0.362 | (-2.44, 0.89) |
| % elderly in an institution | -0.10 | 0.47 | 0.823 | (-1.02, 0.81) |
| Admission type (Ref = Emergency) |  |  |  |  |
| Urgent | 0.02 | 0.07 | 0.775 | (-0.12, 0.16) |
| Elective | 0.22 | 0.08 | 0.005 | (0.07, 0.37) |
| Newborn | -0.42 | 0.28 | 0.135 | (-0.96, 0.13) |
| Diagnosis codes | Included | Included | Included | Included |
| Constant | 1.11 | 1.24 | 0.370 | (-1.32, 3.54) |
|  |  |  |  |  |
| Number of observations: 7,159  Log pseudolikelihood: -4,280.06  Pseudo R^2^: 0.025 | | | | |
