# Supplementary material for: Trends in the quality and cost of inpatient surgical procedures in the United States, 2002–2015
Source: PLoS One. 2021 Nov 3;16(11):e0259011. doi: 10.1371/journal.pone.0259011 (PMC8565758; doi:10.1371/journal.pone.0259011)
Supplement: S4 Table — (A) Regression results for cost of CCS 43 heart valve procedures on a year indicator. (B) Regression results for quality of CCS 43 heart valve procedures on a year indicator. (DOCX) [file pone.0259011.s004.docx]

**S11 Table.** Regression Results for Cost and Quality of CCS 43 Heart Valve Procedures on a Year Indicator

S11A Table. Regression results for cost of CCS 43 heart valve procedures on a year indicator

| Cost of CCS 43 | Coefficient | Robust standard error | P-value | 95% confidence interval |
| --- | --- | --- | --- | --- |
| Year 2015 | -3.79 | 0.79 | < 0.001 | (-5.34, -2.24) |
| Age | -0.06 | 0.02 | 0.006 | (-0.10, -0.02) |
| Race (Ref = White) |  |  |  |  |
| Black | 2.48 | 1.04 | 0.018 | (0.43, 4.53) |
| Asian | 1.70 | 1.11 | 0.126 | (-0.48, 3.87) |
| Hispanic | -0.46 | 1.23 | 0.710 | (-2.87, 1.96) |
| Female | -0.23 | 0.28 | 0.415 | (-0.77, 0.32) |
| Number of Charlson-Deyo comorbidity (Ref = 0) |  |  |  |  |
| 1 | 0.10 | 0.35 | 0.767 | (-0.58, 0.79) |
| 2 | -0.37 | 0.45 | 0.409 | (-1.24, 0.51) |
| 3 | -0.11 | 0.64 | 0.859 | (-1.36, 1.14) |
| 4 | -0.75 | 0.97 | 0.438 | (-2.65, 1.15) |
| 5 | -2.01 | 2.34 | 0.392 | (-6.60, 2.59) |
| Teaching hospital | 1.04 | 0.82 | 0.205 | (-0.57, 2.65) |
| Transferred from other hospitals | -0.73 | 0.66 | 0.268 | (-2.01, 0.56) |
| Transferred to other hospitals | 4.05 | 1.28 | 0.002 | (1.53, 6.56) |
| Social Characteristics |  |  |  |  |
| % urban in the community | 0.22 | 0.75 | 0.768 | (-1.25, 1.69) |
| % of the employed in the community | -19.51 | 10.10 | 0.054 | (-39.33, 0.31) |
| % Hispanic in the community | 3.67 | 3.04 | 0.227 | (-2.29, 9.63) |
| % single in the community | 5.19 | 4.44 | 0.243 | (-3.53, 13.90) |
| % of the poor in the community | -2.82 | 7.68 | 0.713 | (-17.89, 12.24) |
| Social Security income | -0.11 | 0.21 | 0.610 | (-0.51, 0.30) |
| Median household income | 0.02 | 0.02 | 0.263 | (-0.02, 0.06) |
| % with education less than high school | -2.07 | 4.14 | 0.617 | (-10.20, 6.06) |
| % sensory disability among elderly | -1.56 | 4.68 | 0.738 | (-10.74, 7.61) |
| % non-institutionalized elderly with physical disability | 9.35 | 4.00 | 0.020 | (1.49, 17.20) |
| % people with mental disability in the community | -2.36 | 4.70 | 0.616 | (-11.59, 6.87) |
| % people with self-care disability | -8.76 | 5.90 | 0.138 | (-20.34, 2.82) |
| % people with difficulty going-outside-the-home disability | -0.76 | 4.44 | 0.864 | (-9.47, 7.95) |
| % elderly in an institution | -6.29 | 2.95 | 0.033 | (-12.07, -0.51) |
| Admission type (Ref = Emergency) |  |  |  |  |
| Urgent | -1.66 | 0.93 | 0.075 | (-3.49, 0.17) |
| Elective | -5.20 | 0.67 | < 0.001 | (-6.52, -3.87) |
| Newborn | 0.12 | 6.53 | 0.985 | (-12.69, 12.94) |
| Diagnosis codes | Included | Included | Included | Included |
| Constant | 84.21 | 10.16 | < 0.001 | (64.28, 104.13) |
|  |  |  |  |  |
| Number of observations: 14,878  R-squared: 0.07  Root MSE: 15.52 | | | | |

S11B Table. Regression results for quality of CCS 43 heart valve procedures on a year indicator

| Quality of CCS 43 | Coefficient | Robust standard error | P-value | 95% confidence interval |
| --- | --- | --- | --- | --- |
| Year 2015 | 0.42 | 0.05 | < 0.001 | (0.33, 0.51) |
| Age | -0.02 | 0.00 | < 0.001 | (-0.02, -0.01) |
| Race (Ref = White) |  |  |  |  |
| Black | -0.17 | 0.11 | 0.106 | (-0.38, 0.04) |
| Asian | -0.01 | 0.14 | 0.936 | (-0.28, 0.26) |
| Hispanic | -0.38 | 0.16 | 0.020 | (-0.70, -0.06) |
| Female | -0.21 | 0.04 | < 0.001 | (-0.29, -0.14) |
| Number of Charlson-Deyo comorbidity (Ref = 0) |  |  |  |  |
| 1 | -0.21 | 0.05 | < 0.001 | (-0.31, -0.11) |
| 2 | -0.29 | 0.06 | < 0.001 | (-0.40, -0.18) |
| 3 | -0.29 | 0.09 | 0.001 | (-0.46, -0.12) |
| 4 | -0.19 | 0.18 | 0.294 | (-0.56, 0.17) |
| 5 | -0.90 | 0.47 | 0.058 | (-1.82, 0.03) |
| Teaching hospital | 0.02 | 0.04 | 0.549 | (-0.05, 0.10) |
| Transferred from other hospitals | -0.33 | 0.07 | < 0.001 | (-0.47, -0.19) |
| Transferred to other hospitals | 0.03 | 0.12 | 0.806 | (-0.20, 0.26) |
| Social Characteristics |  |  |  |  |
| % urban in the community | -0.14 | 0.09 | 0.092 | (-0.31, 0.02) |
| % of the employed in the community | -0.31 | 0.98 | 0.754 | (-2.23, 1.62) |
| % Hispanic in the community | 0.23 | 0.20 | 0.240 | (-0.15, 0.62) |
| % single in the community | 0.11 | 0.39 | 0.787 | (-0.66, 0.88) |
| % of the poor in the community | -0.03 | 0.59 | 0.958 | (-1.19, 1.13) |
| Social Security income | 0.00 | 0.03 | 0.975 | (-0.05, 0.05) |
| Median household income | 0.00 | 0.00 | 0.642 | (0.00, 0.00) |
| % with education less than high school | -0.02 | 0.40 | 0.960 | (-0.80, 0.76) |
| % sensory disability among elderly | 0.14 | 0.61 | 0.823 | (-1.05, 1.33) |
| % non-institutionalized elderly with physical disability | -0.78 | 0.48 | 0.103 | (-1.73, 0.16) |
| % people with mental disability in the community | -1.25 | 0.67 | 0.062 | (-2.57, 0.07) |
| % people with self-care disability | 0.59 | 0.86 | 0.495 | (-1.10, 2.27) |
| % people with difficulty going-outside-the-home disability | 0.10 | 0.58 | 0.862 | (-1.03, 1.23) |
| % elderly in an institution | -0.05 | 0.36 | 0.895 | (-0.75, 0.66) |
| Admission type (Ref = Emergency) |  |  |  |  |
| Urgent | 0.10 | 0.07 | 0.150 | (-0.03, 0.23) |
| Elective | 0.26 | 0.06 | < 0.001 | (0.15, 0.38) |
| Newborn | 0.01 | 0.43 | 0.978 | (-0.83, 0.85) |
| Diagnosis codes | Included | Included | Included | Included |
| Constant | 2.50 | 1.15 | 0.030 | (0.25, 4.76) |
|  |  |  |  |  |
| Number of observations: 14,878  Log pseudolikelihood: -7,667.96  Pseudo R^2^: 0.028 | | | | |
