# Supplementary material for: Trends in the quality and cost of inpatient surgical procedures in the United States, 2002–2015
Source: PLoS One. 2021 Nov 3;16(11):e0259011. doi: 10.1371/journal.pone.0259011 (PMC8565758; doi:10.1371/journal.pone.0259011)
Supplement: S5 Table — (A) Regression results for cost of CCS 44 coronary artery bypass grafting on a year indicator. (B) Regression results for quality of CCS 44 coronary artery bypass grafting on a year indicator. (DOCX) [file pone.0259011.s005.docx]

**S12 Table.** Regression Results for Cost and Quality of CCS 44 Coronary Artery Bypass Grafting on a Year Indicator

S12A Table. Regression results for cost of CCS 44 coronary artery bypass grafting on a year indicator

| Cost of CCS 44 | Coefficient | Robust standard error | P-value | 95% confidence interval |
| --- | --- | --- | --- | --- |
| Year 2015 | 3.30 | 0.54 | < 0.001 | (2.24, 4.35) |
| Age | 0.06 | 0.01 | < 0.001 | (0.03, 0.08) |
| Race (Ref = White) |  |  |  |  |
| Black | 0.31 | 0.47 | 0.513 | (-0.62, 1.23) |
| Asian | 2.39 | 0.69 | 0.001 | (1.03, 3.75) |
| Hispanic | 0.48 | 0.72 | 0.501 | (-0.93, 1.90) |
| Female | 0.32 | 0.13 | 0.013 | (0.07, 0.58) |
| Number of Charlson-Deyo comorbidity (Ref = 0) |  |  |  |  |
| 1 | 0.67 | 0.17 | < 0.001 | (0.35, 1.00) |
| 2 | 1.03 | 0.19 | < 0.001 | (0.65, 1.41) |
| 3 | 1.47 | 0.28 | < 0.001 | (0.93, 2.02) |
| 4 | 1.42 | 0.44 | 0.001 | (0.55, 2.28) |
| 5 | 1.55 | 1.21 | 0.200 | (-0.82, 3.93) |
| Teaching hospital | -0.79 | 0.60 | 0.187 | (-1.95, 0.38) |
| Transferred from other hospitals | -0.10 | 0.46 | 0.835 | (-0.99, 0.80) |
| Transferred to other hospitals | 2.42 | 0.70 | 0.001 | (1.04, 3.80) |
| Social Characteristics |  |  |  |  |
| % urban in the community | -0.80 | 0.53 | 0.128 | (-1.83, 0.23) |
| % of the employed in the community | -14.62 | 6.22 | 0.019 | (-26.83, -2.41) |
| % Hispanic in the community | 7.05 | 2.27 | 0.002 | (2.60, 11.50) |
| % single in the community | 7.15 | 3.43 | 0.038 | (0.41, 13.88) |
| % of the poor in the community | -3.69 | 4.74 | 0.436 | (-12.98, 5.60) |
| Social Security income | -0.04 | 0.15 | 0.787 | (-0.34, 0.26) |
| Median household income | 0.03 | 0.02 | 0.195 | (-0.01, 0.07) |
| % with education less than high school | -3.95 | 3.25 | 0.224 | (-10.32, 2.42) |
| % sensory disability among elderly | 0.72 | 2.84 | 0.801 | (-4.86, 6.29) |
| % non-institutionalized elderly with physical disability | 3.29 | 2.52 | 0.193 | (-1.67, 8.24) |
| % people with mental disability in the community | -0.66 | 3.38 | 0.846 | (-7.28, 5.97) |
| % people with self-care disability | -1.33 | 3.98 | 0.739 | (-9.13, 6.48) |
| % people with difficulty going-outside-the-home disability | -3.16 | 2.45 | 0.197 | (-7.96, 1.64) |
| % elderly in an institution | -1.13 | 1.81 | 0.534 | (-4.69, 2.43) |
| Admission type (Ref = Emergency) |  |  |  |  |
| Urgent | -0.40 | 0.46 | 0.384 | (-1.30, 0.50) |
| Elective | -1.88 | 0.36 | < 0.001 | (-2.58, -1.18) |
| Newborn | -2.25 | 2.27 | 0.322 | (-6.71, 2.21) |
| Diagnosis codes | Included | Included | Included | Included |
| Constant | 56.62 | 6.74 | < 0.001 | (43.40, 69.84) |
|  |  |  |  |  |
| Number of observations: 30,978  R-squared: 0.05  Root MSE: 10.78 | | | | |

S12B Table. Regression results for quality of CCS 44 coronary artery bypass grafting on a year indicator

| Quality of CCS 44 | Coefficient | Robust standard error | P-value | 95% confidence interval |
| --- | --- | --- | --- | --- |
| Year 2015 | 0.28 | 0.04 | < 0.001 | (0.21, 0.36) |
| Age | -0.03 | 0.00 | < 0.001 | (-0.04, -0.03) |
| Race (Ref = White) |  |  |  |  |
| Black | -0.08 | 0.07 | 0.252 | (-0.22, 0.06) |
| Asian | -0.21 | 0.09 | 0.027 | (-0.39, -0.02) |
| Hispanic | -0.11 | 0.12 | 0.338 | (-0.34, 0.12) |
| Female | -0.29 | 0.03 | < 0.001 | (-0.36, -0.23) |
| Number of Charlson-Deyo comorbidity (Ref = 0) |  |  |  |  |
| 1 | -0.20 | 0.04 | < 0.001 | (-0.28, -0.12) |
| 2 | -0.46 | 0.04 | < 0.001 | (-0.55, -0.37) |
| 3 | -0.55 | 0.06 | < 0.001 | (-0.66, -0.44) |
| 4 | -0.74 | 0.10 | < 0.001 | (-0.93, -0.55) |
| 5 | -1.31 | 0.25 | < 0.001 | (-1.80, -0.82) |
| Teaching hospital | -0.01 | 0.03 | 0.795 | (-0.07, 0.05) |
| Transferred from other hospitals | -0.10 | 0.04 | 0.016 | (-0.18, -0.02) |
| Transferred to other hospitals | -0.05 | 0.09 | 0.526 | (-0.22, 0.11) |
| Social Characteristics |  |  |  |  |
| % urban in the community | 0.03 | 0.06 | 0.618 | (-0.08, 0.14) |
| % of the employed in the community | -0.75 | 0.68 | 0.268 | (-2.08, 0.58) |
| % Hispanic in the community | -0.04 | 0.14 | 0.768 | (-0.31, 0.23) |
| % single in the community | -0.43 | 0.28 | 0.125 | (-0.97, 0.12) |
| % of the poor in the community | 0.00 | 0.40 | 0.998 | (-0.78, 0.78) |
| Social Security income | -0.01 | 0.02 | 0.377 | (-0.04, 0.02) |
| Median household income | 0.00 | 0.00 | 0.436 | (0.00, 0.00) |
| % with education less than high school | -0.40 | 0.26 | 0.127 | (-0.92, 0.11) |
| % sensory disability among elderly | 0.83 | 0.44 | 0.060 | (-0.04, 1.69) |
| % non-institutionalized elderly with physical disability | -0.22 | 0.37 | 0.556 | (-0.95, 0.51) |
| % people with mental disability in the community | -0.10 | 0.48 | 0.835 | (-1.03, 0.83) |
| % people with self-care disability | -0.49 | 0.59 | 0.412 | (-1.65, 0.68) |
| % people with difficulty going-outside-the-home disability | -0.54 | 0.42 | 0.204 | (-1.37, 0.29) |
| % elderly in an institution | 0.26 | 0.27 | 0.337 | (-0.27, 0.78) |
| Admission type (Ref = Emergency) |  |  |  |  |
| Urgent | 0.01 | 0.04 | 0.843 | (-0.07, 0.09) |
| Elective | 0.18 | 0.04 | < 0.001 | (0.11, 0.26) |
| Newborn | -0.19 | 0.21 | 0.368 | (-0.61, 0.23) |
| Diagnosis codes | Included | Included | Included | Included |
| Constant | 5.16 | 0.78 | < 0.001 | (3.64, 6.68) |
|  |  |  |  |  |
| Number of observations: 30,978  Log pseudolikelihood: -14,462.12  Pseudo R^2^: 0.028 | | | | |
