# Supplementary material for: Trends in the quality and cost of inpatient surgical procedures in the United States, 2002–2015
Source: PLoS One. 2021 Nov 3;16(11):e0259011. doi: 10.1371/journal.pone.0259011 (PMC8565758; doi:10.1371/journal.pone.0259011)
Supplement: S6 Table — (A) Regression results for cost of CCS 45 percutaneous transluminal coronary angioplasty on a year indicator. (B) Regression results for quality of CCS 45 percutaneous transluminal coronary angioplasty on a year indicator. (DOCX) [file pone.0259011.s006.docx]

**S13 Table.** Regression Results for Cost and Quality of CCS 45 Percutaneous Transluminal Coronary Angioplasty on a Year Indicator

S13A Table. Regression results for cost of CCS 45 percutaneous transluminal coronary angioplasty on a year indicator

| Cost of CCS 45 | Coefficient | Robust standard error | P-value | 95% confidence interval |
| --- | --- | --- | --- | --- |
| Year 2015 | 2.89 | 0.23 | < 0.001 | (2.44, 3.34) |
| Age | 0.02 | 0.00 | < 0.001 | (0.01, 0.03) |
| Race (Ref = White) |  |  |  |  |
| Black | 0.31 | 0.17 | 0.072 | (-0.03, 0.64) |
| Asian | 0.17 | 0.29 | 0.546 | (-0.39, 0.73) |
| Hispanic | 0.55 | 0.25 | 0.028 | (0.06, 1.04) |
| Female | 0.16 | 0.05 | 0.001 | (0.06, 0.25) |
| Number of Charlson-Deyo comorbidity (Ref = 0) |  |  |  |  |
| 1 | 0.24 | 0.06 | < 0.001 | (0.11, 0.37) |
| 2 | 0.63 | 0.08 | < 0.001 | (0.47, 0.80) |
| 3 | 1.13 | 0.12 | < 0.001 | (0.90, 1.36) |
| 4 | 1.16 | 0.18 | < 0.001 | (0.81, 1.52) |
| 5 | 0.88 | 0.39 | 0.024 | (0.12, 1.65) |
| Teaching hospital | 0.00 | 0.25 | 0.997 | (-0.48, 0.48) |
| Transferred from other hospitals | -0.15 | 0.19 | 0.425 | (-0.52, 0.22) |
| Transferred to other hospitals | 1.84 | 0.37 | < 0.001 | (1.12, 2.56) |
| Social Characteristics |  |  |  |  |
| % urban in the community | -0.36 | 0.20 | 0.078 | (-0.75, 0.04) |
| % of the employed in the community | 2.54 | 2.12 | 0.231 | (-1.61, 6.69) |
| % Hispanic in the community | 1.97 | 0.89 | 0.026 | (0.23, 3.71) |
| % single in the community | -1.00 | 1.39 | 0.472 | (-3.72, 1.72) |
| % of the poor in the community | 1.53 | 1.80 | 0.395 | (-2.01, 5.07) |
| Social Security income | -0.25 | 0.07 | < 0.001 | (-0.38, -0.11) |
| Median household income | -0.01 | 0.01 | 0.307 | (-0.02, 0.01) |
| % with education less than high school | -3.72 | 1.37 | 0.007 | (-6.41, -1.03) |
| % sensory disability among elderly | 0.72 | 1.03 | 0.487 | (-1.31, 2.74) |
| % non-institutionalized elderly with physical disability | 0.68 | 1.02 | 0.502 | (-1.31, 2.68) |
| % people with mental disability in the community | 1.73 | 1.49 | 0.246 | (-1.19, 4.66) |
| % people with self-care disability | -1.24 | 1.60 | 0.439 | (-4.38, 1.90) |
| % people with difficulty going-outside-the-home disability | -1.38 | 1.07 | 0.197 | (-3.46, 0.71) |
| % elderly in an institution | 1.39 | 0.71 | 0.049 | (0.00, 2.78) |
| Admission type (Ref = Emergency) |  |  |  |  |
| Urgent | -0.38 | 0.18 | 0.032 | (-0.73, -0.03) |
| Elective | -1.02 | 0.18 | < 0.001 | (-1.38, -0.67) |
| Newborn | -0.37 | 0.91 | 0.683 | (-2.16, 1.41) |
| Diagnosis codes | Included | Included | Included | Included |
| Constant | 22.39 | 2.44 | < 0.001 | (17.61, 27.17) |
|  |  |  |  |  |
| Number of observations: 63,620  R-squared: 0.13  Root MSE: 5.24 | | | | |

S13B Table. Regression results for quality of CCS 45 percutaneous transluminal coronary angioplasty on a year indicator

| Quality of CCS 45 | Coefficient | Robust standard error | P-value | 95% confidence interval |
| --- | --- | --- | --- | --- |
| Year 2015 | 0.09 | 0.03 | 0.003 | (0.03, 0.15) |
| Age | -0.03 | 0.00 | < 0.001 | (-0.04, -0.03) |
| Race (Ref = White) |  |  |  |  |
| Black | -0.17 | 0.05 | 0.001 | (-0.27, -0.07) |
| Asian | -0.12 | 0.08 | 0.129 | (-0.27, 0.03) |
| Hispanic | 0.04 | 0.10 | 0.692 | (-0.16, 0.24) |
| Female | -0.23 | 0.02 | < 0.001 | (-0.28, -0.18) |
| Number of Charlson-Deyo comorbidity (Ref = 0) |  |  |  |  |
| 1 | -0.26 | 0.04 | < 0.001 | (-0.34, -0.19) |
| 2 | -0.67 | 0.04 | < 0.001 | (-0.75, -0.58) |
| 3 | -0.99 | 0.05 | < 0.001 | (-1.09, -0.90) |
| 4 | -1.05 | 0.07 | < 0.001 | (-1.19, -0.91) |
| 5 | -1.08 | 0.17 | < 0.001 | (-1.40, -0.75) |
| Teaching hospital | -0.02 | 0.02 | 0.319 | (-0.07, 0.02) |
| Transferred from other hospitals | -0.11 | 0.04 | 0.002 | (-0.19, -0.04) |
| Transferred to other hospitals | -0.49 | 0.13 | < 0.001 | (-0.75, -0.23) |
| Social Characteristics |  |  |  |  |
| % urban in the community | 0.03 | 0.05 | 0.499 | (-0.06, 0.12) |
| % of the employed in the community | -0.67 | 0.54 | 0.214 | (-1.73, 0.39) |
| % Hispanic in the community | -0.07 | 0.11 | 0.521 | (-0.29, 0.15) |
| % single in the community | -0.36 | 0.22 | 0.105 | (-0.79, 0.07) |
| % of the poor in the community | -0.45 | 0.34 | 0.184 | (-1.11, 0.21) |
| Social Security income | 0.00 | 0.01 | 0.773 | (-0.03, 0.02) |
| Median household income | 0.00 | 0.00 | 0.591 | (0.00, 0.00) |
| % with education less than high school | -0.43 | 0.23 | 0.061 | (-0.87, 0.02) |
| % sensory disability among elderly | 0.18 | 0.36 | 0.621 | (-0.53, 0.89) |
| % non-institutionalized elderly with physical disability | -0.46 | 0.29 | 0.116 | (-1.04, 0.11) |
| % people with mental disability in the community | 0.16 | 0.44 | 0.708 | (-0.70, 1.03) |
| % people with self-care disability | 0.56 | 0.50 | 0.260 | (-0.42, 1.54) |
| % people with difficulty going-outside-the-home disability | -0.12 | 0.35 | 0.735 | (-0.80, 0.56) |
| % elderly in an institution | 0.21 | 0.22 | 0.354 | (-0.23, 0.65) |
| Admission type (Ref = Emergency) |  |  |  |  |
| Urgent | 0.15 | 0.03 | < 0.001 | (0.08, 0.22) |
| Elective | 0.28 | 0.04 | < 0.001 | (0.21, 0.35) |
| Newborn | -0.10 | 0.17 | 0.560 | (-0.44, 0.24) |
| Diagnosis codes | Included | Included | Included | Included |
| Constant | 5.43 | 0.62 | < 0.001 | (4.21, 6.65) |
|  |  |  |  |  |
| Number of observations: 63,620  Log pseudolikelihood: -23,507.12  Pseudo R^2^: 0.049 | | | | |
