# Supplementary material for: Trends in the quality and cost of inpatient surgical procedures in the United States, 2002–2015
Source: PLoS One. 2021 Nov 3;16(11):e0259011. doi: 10.1371/journal.pone.0259011 (PMC8565758; doi:10.1371/journal.pone.0259011)
Supplement: S7 Table — (A) Regression results for cost of CCS 71 gastrostomy on a year indicator. (B) Regression results for quality of CCS 71 gastrostomy on a year indicator. (DOCX) [file pone.0259011.s007.docx]

**S14 Table.** Regression Results for Cost and Quality of CCS 71 Gastrostomy on a Year Indicator

S14A Table. Regression results for cost of CCS 71 gastrostomy on a year indicator

| Cost of CCS 71 | Coefficient | Robust standard error | P-value | 95% confidence interval |
| --- | --- | --- | --- | --- |
| Year 2015 | 1.05 | 0.32 | 0.001 | (0.43, 1.67) |
| Age | -0.05 | 0.01 | < 0.001 | (-0.07, -0.03) |
| Race (Ref = White) |  |  |  |  |
| Black | -0.32 | 0.24 | 0.186 | (-0.80, 0.16) |
| Asian | 0.20 | 0.47 | 0.681 | (-0.74, 1.13) |
| Hispanic | -1.05 | 0.44 | 0.017 | (-1.90, -0.19) |
| Female | -0.69 | 0.15 | < 0.001 | (-0.99, -0.39) |
| Number of Charlson-Deyo comorbidity (Ref = 0) |  |  |  |  |
| 1 | 0.32 | 0.22 | 0.147 | (-0.11, 0.75) |
| 2 | 0.60 | 0.23 | 0.007 | (0.16, 1.05) |
| 3 | 0.54 | 0.30 | 0.069 | (-0.04, 1.13) |
| 4 | 0.70 | 0.42 | 0.097 | (-0.13, 1.53) |
| 5 | -0.93 | 0.79 | 0.238 | (-2.48, 0.62) |
| Teaching hospital | 1.15 | 0.28 | < 0.001 | (0.59, 1.70) |
| Transferred from other hospitals | 2.76 | 0.63 | < 0.001 | (1.52, 3.99) |
| Transferred to other hospitals | 0.91 | 0.41 | 0.027 | (0.10, 1.72) |
| Social Characteristics |  |  |  |  |
| % urban in the community | -0.70 | 0.40 | 0.082 | (-1.49, 0.09) |
| % of the employed in the community | 2.13 | 3.67 | 0.561 | (-5.06, 9.32) |
| % Hispanic in the community | 2.58 | 0.93 | 0.006 | (0.74, 4.41) |
| % single in the community | 10.45 | 2.13 | < 0.001 | (6.27, 14.62) |
| % of the poor in the community | 5.84 | 2.63 | 0.027 | (0.68, 11.00) |
| Social Security income | 0.15 | 0.11 | 0.183 | (-0.07, 0.37) |
| Median household income | 0.10 | 0.01 | < 0.001 | (0.08, 0.13) |
| % with education less than high school | -0.67 | 1.80 | 0.707 | (-4.20, 2.85) |
| % sensory disability among elderly | 0.43 | 2.25 | 0.848 | (-3.99, 4.85) |
| % non-institutionalized elderly with physical disability | -5.13 | 2.08 | 0.014 | (-9.21, -1.05) |
| % people with mental disability in the community | 3.25 | 2.66 | 0.222 | (-1.97, 8.47) |
| % people with self-care disability | 2.76 | 3.27 | 0.398 | (-3.65, 9.17) |
| % people with difficulty going-outside-the-home disability | 2.46 | 2.29 | 0.284 | (-2.04, 6.95) |
| % elderly in an institution | -2.23 | 1.44 | 0.122 | (-5.07, 0.60) |
| Admission type (Ref = Emergency) |  |  |  |  |
| Urgent | -0.40 | 0.28 | 0.152 | (-0.96, 0.15) |
| Elective | -1.06 | 0.33 | 0.001 | (-1.71, -0.41) |
| Newborn | -0.09 | 2.09 | 0.967 | (-4.19, 4.02) |
| Diagnosis codes | Included | Included | Included | Included |
| Constant | 11.29 | 4.29 | 0.009 | (2.88, 19.71) |
|  |  |  |  |  |
| Number of observations: 13,851  R-squared: 0.10  Root MSE: 8.56 | | | | |

S14B Table. Regression results for quality of CCS 71 gastrostomy on a year indicator

| Quality of CCS 71 | Coefficient | Robust standard error | P-value | 95% confidence interval |
| --- | --- | --- | --- | --- |
| Year 2015 | 0.12 | 0.04 | 0.005 | (0.03, 0.20) |
| Age | -0.01 | 0.00 | < 0.001 | (-0.02, -0.01) |
| Race (Ref = White) |  |  |  |  |
| Black | 0.00 | 0.05 | 0.965 | (-0.10, 0.10) |
| Asian | 0.05 | 0.09 | 0.556 | (-0.12, 0.23) |
| Hispanic | 0.07 | 0.10 | 0.509 | (-0.13, 0.27) |
| Female | 0.19 | 0.04 | < 0.001 | (0.12, 0.27) |
| Number of Charlson-Deyo comorbidity (Ref = 0) |  |  |  |  |
| 1 | -0.19 | 0.05 | < 0.001 | (-0.28, -0.09) |
| 2 | -0.32 | 0.05 | < 0.001 | (-0.43, -0.22) |
| 3 | -0.51 | 0.07 | < 0.001 | (-0.64, -0.37) |
| 4 | -0.59 | 0.11 | < 0.001 | (-0.80, -0.38) |
| 5 | -0.68 | 0.28 | 0.015 | (-1.23, -0.13) |
| Teaching hospital | 0.03 | 0.03 | 0.311 | (-0.03, 0.10) |
| Transferred from other hospitals | -0.16 | 0.10 | 0.096 | (-0.36, 0.03) |
| Transferred to other hospitals | 0.43 | 0.09 | < 0.001 | (0.25, 0.61) |
| Social Characteristics |  |  |  |  |
| % urban in the community | -0.05 | 0.07 | 0.516 | (-0.19, 0.10) |
| % of the employed in the community | -0.04 | 0.72 | 0.958 | (-1.45, 1.38) |
| % Hispanic in the community | 0.13 | 0.13 | 0.319 | (-0.12, 0.38) |
| % single in the community | -0.11 | 0.32 | 0.718 | (-0.73, 0.50) |
| % of the poor in the community | -0.38 | 0.43 | 0.383 | (-1.22, 0.47) |
| Social Security income | 0.01 | 0.02 | 0.751 | (-0.03, 0.04) |
| Median household income | 0.00 | 0.00 | 0.747 | (0.00, 0.00) |
| % with education less than high school | -0.29 | 0.32 | 0.352 | (-0.91, 0.32) |
| % sensory disability among elderly | 0.42 | 0.53 | 0.424 | (-0.62, 1.46) |
| % non-institutionalized elderly with physical disability | -0.45 | 0.41 | 0.277 | (-1.26, 0.36) |
| % people with mental disability in the community | 0.59 | 0.54 | 0.275 | (-0.47, 1.64) |
| % people with self-care disability | 0.38 | 0.72 | 0.593 | (-1.02, 1.79) |
| % people with difficulty going-outside-the-home disability | 0.01 | 0.50 | 0.982 | (-0.96, 0.98) |
| % elderly in an institution | 0.45 | 0.30 | 0.137 | (-0.14, 1.04) |
| Admission type (Ref = Emergency) |  |  |  |  |
| Urgent | 0.05 | 0.04 | 0.255 | (-0.04, 0.14) |
| Elective | 0.06 | 0.06 | 0.356 | (-0.07, 0.18) |
| Newborn | 0.03 | 0.26 | 0.898 | (-0.48, 0.55) |
| Diagnosis codes | Included | Included | Included | Included |
| Constant | 1.50 | 0.85 | 0.077 | (-0.16, 3.16) |
|  |  |  |  |  |
| Number of observations: 13,851  Log pseudolikelihood: -9,227.94  Pseudo R^2^: 0.02 | | | | |
