# Supplementary material for: Trends in the quality and cost of inpatient surgical procedures in the United States, 2002–2015
Source: PLoS One. 2021 Nov 3;16(11):e0259011. doi: 10.1371/journal.pone.0259011 (PMC8565758; doi:10.1371/journal.pone.0259011)
Supplement: S8 Table — (A) Regression results for cost of CCS 73 ileostomy and other enterostomy on a year indicator. (B) Regression results for quality of CCS 73 ileostomy and other enterostomy on a year indicator. (DOCX) [file pone.0259011.s008.docx]

**S15 Table.** Regression Results for Cost and Quality of CCS 73 Ileostomy and Other Enterostomy on a Year Indicator

S15A Table. Regression results for cost of CCS 73 ileostomy and other enterostomy on a year indicator

| Cost of CCS 73 | Coefficient | Robust standard error | P-value | 95% confidence interval |
| --- | --- | --- | --- | --- |
| Year 2015 | -2.02 | 2.07 | 0.330 | (-6.09, 2.05) |
| Age | -0.18 | 0.11 | 0.089 | (-0.39, 0.03) |
| Race (Ref = White) |  |  |  |  |
| Black | -3.91 | 3.39 | 0.248 | (-10.57, 2.74) |
| Asian | -5.25 | 4.09 | 0.199 | (-13.29, 2.78) |
| Hispanic | 4.16 | 5.97 | 0.486 | (-7.57, 15.90) |
| Female | -0.67 | 1.81 | 0.713 | (-4.23, 2.89) |
| Number of Charlson-Deyo comorbidity (Ref = 0) |  |  |  |  |
| 1 | -1.59 | 2.24 | 0.479 | (-5.99, 2.82) |
| 2 | -1.93 | 2.69 | 0.473 | (-7.22, 3.35) |
| 3 | 1.87 | 3.72 | 0.616 | (-5.44, 9.18) |
| 4 | -8.98 | 4.38 | 0.041 | (-17.57, -0.38) |
| Teaching hospital | 2.74 | 1.90 | 0.150 | (-0.99, 6.46) |
| Transferred from other hospitals | 12.84 | 7.30 | 0.079 | (-1.50, 27.18) |
| Transferred to other hospitals | 0.14 | 4.07 | 0.972 | (-7.85, 8.13) |
| Social Characteristics |  |  |  |  |
| % urban in the community | -2.01 | 3.68 | 0.585 | (-9.23, 5.22) |
| % of the employed in the community | -22.17 | 51.97 | 0.670 | (-124.25, 79.90) |
| % Hispanic in the community | 5.16 | 6.00 | 0.390 | (-6.62, 16.94) |
| % single in the community | 42.67 | 21.85 | 0.051 | (-0.26, 85.59) |
| % of the poor in the community | -14.83 | 23.80 | 0.533 | (-61.57, 31.91) |
| Social Security income | -0.55 | 0.81 | 0.497 | (-2.15, 1.04) |
| Median household income | 0.20 | 0.11 | 0.072 | (-0.02, 0.43) |
| % with education less than high school | -9.14 | 17.15 | 0.594 | (-42.82, 24.54) |
| % sensory disability among elderly | 32.98 | 25.15 | 0.190 | (-16.42, 82.38) |
| % non-institutionalized elderly with physical disability | 5.03 | 14.39 | 0.727 | (-23.23, 33.29) |
| % people with mental disability in the community | 4.26 | 28.85 | 0.883 | (-52.40, 60.93) |
| % people with self-care disability | -31.20 | 49.66 | 0.530 | (-128.75, 66.34) |
| % people with difficulty going-outside-the-home disability | 13.60 | 34.35 | 0.692 | (-53.87, 81.08) |
| % elderly in an institution | -33.69 | 13.29 | 0.012 | (-59.79, -7.58) |
| Admission type (Ref = Emergency) |  |  |  |  |
| Urgent | -5.28 | 2.23 | 0.018 | (-9.66, -0.90) |
| Elective | -4.85 | 2.20 | 0.028 | (-9.18, -0.52) |
| Newborn | -10.19 | 5.01 | 0.043 | (-20.04, -0.35) |
| Diagnosis codes | Included | Included | Included | Included |
| Constant | 51.93 | 61.22 | 0.397 | (-68.32, 172.19) |
|  |  |  |  |  |
| Number of observations: 726  R-squared: 0.16  Root MSE: 22.91 | | | | |

S15B Table. Regression results for quality of CCS 73 ileostomy and other enterostomy on a year indicator

| Quality of CCS 73 | Coefficient | Robust standard error | P-value | 95% confidence interval |
| --- | --- | --- | --- | --- |
| Year 2015 | -0.07 | 0.18 | 0.694 | (-0.43, 0.29) |
| Age | -0.04 | 0.01 | 0.001 | (-0.06, -0.01) |
| Race (Ref = White) |  |  |  |  |
| Black | -0.33 | 0.28 | 0.232 | (-0.88, 0.21) |
| Asian | -0.59 | 0.45 | 0.194 | (-1.48, 0.30) |
| Hispanic | -0.77 | 0.57 | 0.176 | (-1.89, 0.35) |
| Female | -0.03 | 0.16 | 0.835 | (-0.35, 0.28) |
| Number of Charlson-Deyo comorbidity (Ref = 0) |  |  |  |  |
| 1 | -0.08 | 0.22 | 0.710 | (-0.51, 0.35) |
| 2 | 0.14 | 0.26 | 0.588 | (-0.36, 0.64) |
| 3 | 0.39 | 0.33 | 0.231 | (-0.25, 1.04) |
| 4 | -0.19 | 0.57 | 0.745 | (-1.31, 0.94) |
| Teaching hospital | 0.10 | 0.14 | 0.484 | (-0.18, 0.38) |
| Transferred from other hospitals | -0.06 | 0.42 | 0.883 | (-0.89, 0.77) |
| Transferred to other hospitals | 1.15 | 0.56 | 0.041 | (0.05, 2.25) |
| Social Characteristics |  |  |  |  |
| % urban in the community | 0.18 | 0.34 | 0.598 | (-0.49, 0.85) |
| % of the employed in the community | 1.52 | 3.27 | 0.641 | (-4.88, 7.92) |
| % Hispanic in the community | 0.07 | 0.58 | 0.899 | (-1.07, 1.22) |
| % single in the community | -0.43 | 1.38 | 0.753 | (-3.13, 2.27) |
| % of the poor in the community | 0.81 | 2.26 | 0.719 | (-3.62, 5.25) |
| Social Security income | 0.00 | 0.09 | 0.989 | (-0.18, 0.17) |
| Median household income | -0.01 | 0.01 | 0.570 | (-0.02, 0.01) |
| % with education less than high school | -0.19 | 1.53 | 0.902 | (-3.19, 2.82) |
| % sensory disability among elderly | 2.35 | 2.60 | 0.367 | (-2.75, 7.44) |
| % non-institutionalized elderly with physical disability | -0.32 | 1.74 | 0.856 | (-3.72, 3.09) |
| % people with mental disability in the community | -4.09 | 2.73 | 0.134 | (-9.44, 1.25) |
| % people with self-care disability | 2.60 | 3.68 | 0.480 | (-4.61, 9.82) |
| % people with difficulty going-outside-the-home disability | 1.44 | 2.39 | 0.548 | (-3.25, 6.12) |
| % elderly in an institution | 1.65 | 1.46 | 0.261 | (-1.22, 4.52) |
| Admission type (Ref = Emergency) |  |  |  |  |
| Urgent | 0.02 | 0.23 | 0.921 | (-0.43, 0.48) |
| Elective | 0.74 | 0.24 | 0.002 | (0.27, 1.21) |
| Newborn | -0.90 | 1.48 | 0.543 | (-3.80, 2.00) |
| Diagnosis codes | Included | Included | Included | Included |
| Constant | 1.14 | 3.71 | 0.757 | (-6.12, 8.41) |
|  |  |  |  |  |
| Number of observations: 726  Log pseudolikelihood: -461.61  Pseudo R^2^: 0.08 | | | | |
