# Supplementary material for: Trends in the quality and cost of inpatient surgical procedures in the United States, 2002–2015
Source: PLoS One. 2021 Nov 3;16(11):e0259011. doi: 10.1371/journal.pone.0259011 (PMC8565758; doi:10.1371/journal.pone.0259011)
Supplement: S9 Table — (A) Regression results for cost of CCS 75 small bowel resection on a year indicator. (B) Regression results for quality of CCS 75 small bowel resection on a year indicator. (DOCX) [file pone.0259011.s009.docx]

**S16 Table.** Regression Results for Cost and Quality of CCS 75 Small Bowel Resection on a Year Indicator

S16A Table. Regression results for cost of CCS 75 small bowel resection on a year indicator

| Cost of CCS 75 | Coefficient | Robust standard error | P-value | 95% confidence interval |
| --- | --- | --- | --- | --- |
| Year 2015 | -4.39 | 1.08 | < 0.001 | (-6.52, -2.26) |
| Age | -0.03 | 0.04 | 0.482 | (-0.12, 0.06) |
| Race (Ref = White) |  |  |  |  |
| Black | 4.59 | 1.51 | 0.002 | (1.63, 7.55) |
| Asian | 3.38 | 3.58 | 0.345 | (-3.64, 10.40) |
| Hispanic | -4.16 | 2.19 | 0.057 | (-8.45, 0.13) |
| Female | -0.22 | 0.74 | 0.764 | (-1.67, 1.23) |
| Number of Charlson-Deyo comorbidity (Ref = 0) |  |  |  |  |
| 1 | 2.73 | 0.90 | 0.002 | (0.97, 4.50) |
| 2 | 2.66 | 1.02 | 0.009 | (0.66, 4.66) |
| 3 | 2.30 | 1.63 | 0.158 | (-0.89, 5.49) |
| 4 | -1.97 | 2.48 | 0.427 | (-6.84, 2.89) |
| Teaching hospital | 2.17 | 1.12 | 0.053 | (-0.03, 4.37) |
| Transferred from other hospitals | 6.86 | 4.03 | 0.089 | (-1.04, 14.77) |
| Transferred to other hospitals | 8.50 | 3.56 | 0.017 | (1.51, 15.49) |
| Social Characteristics |  |  |  |  |
| % urban in the community | -1.27 | 1.63 | 0.436 | (-4.45, 1.92) |
| % of the employed in the community | -17.86 | 16.83 | 0.289 | (-50.87, 15.15) |
| % Hispanic in the community | 2.51 | 3.47 | 0.470 | (-4.29, 9.31) |
| % single in the community | 10.87 | 7.32 | 0.138 | (-3.48, 25.23) |
| % of the poor in the community | 9.50 | 10.03 | 0.344 | (-10.18, 29.17) |
| Social Security income | 0.41 | 0.45 | 0.362 | (-0.47, 1.28) |
| Median household income | 0.11 | 0.04 | 0.014 | (0.02, 0.19) |
| % with education less than high school | 0.88 | 6.87 | 0.898 | (-12.59, 14.35) |
| % sensory disability among elderly | -0.27 | 10.17 | 0.979 | (-20.22, 19.67) |
| % non-institutionalized elderly with physical disability | 12.01 | 9.17 | 0.191 | (-5.98, 29.99) |
| % people with mental disability in the community | -18.67 | 17.64 | 0.290 | (-53.26, 15.92) |
| % people with self-care disability | -4.00 | 12.19 | 0.743 | (-27.91, 19.92) |
| % people with difficulty going-outside-the-home disability | 1.50 | 11.64 | 0.897 | (-21.33, 24.33) |
| % elderly in an institution | -4.59 | 6.95 | 0.509 | (-18.21, 9.04) |
| Admission type (Ref = Emergency) |  |  |  |  |
| Urgent | -0.55 | 1.36 | 0.686 | (-3.21, 2.11) |
| Elective | -5.52 | 1.07 | < 0.001 | (-7.62, -3.41) |
| Newborn | 0.98 | 6.25 | 0.876 | (-11.28, 13.24) |
| Diagnosis codes | Included | Included | Included | Included |
| Constant | 45.04 | 18.53 | 0.015 | (8.70, 81.37) |
|  |  |  |  |  |
| Number of observations: 5,267  R-squared: 0.05  Root MSE: 25.24 | | | | |

S16B Table. Regression results for quality of CCS 75 small bowel resection on a year indicator

| Quality of CCS 75 | Coefficient | Robust standard error | P-value | 95% confidence interval |
| --- | --- | --- | --- | --- |
| Year 2015 | 0.21 | 0.07 | 0.005 | (0.07, 0.36) |
| Age | -0.03 | 0.00 | < 0.001 | (-0.03, -0.02) |
| Race (Ref = White) |  |  |  |  |
| Black | 0.11 | 0.12 | 0.382 | (-0.13, 0.35) |
| Asian | -0.29 | 0.22 | 0.199 | (-0.73, 0.15) |
| Hispanic | -0.49 | 0.30 | 0.104 | (-1.08, 0.10) |
| Female | -0.06 | 0.07 | 0.411 | (-0.19, 0.08) |
| Number of Charlson-Deyo comorbidity (Ref = 0) |  |  |  |  |
| 1 | -0.33 | 0.08 | < 0.001 | (-0.49, -0.18) |
| 2 | -0.64 | 0.09 | < 0.001 | (-0.81, -0.47) |
| 3 | -0.94 | 0.15 | < 0.001 | (-1.23, -0.64) |
| 4 | -1.04 | 0.36 | 0.004 | (-1.75, -0.33) |
| Teaching hospital | -0.13 | 0.06 | 0.040 | (-0.26, -0.01) |
| Transferred from other hospitals | -0.22 | 0.16 | 0.157 | (-0.53, 0.09) |
| Transferred to other hospitals | 0.25 | 0.22 | 0.267 | (-0.19, 0.68) |
| Social Characteristics |  |  |  |  |
| % urban in the community | 0.15 | 0.13 | 0.259 | (-0.11, 0.41) |
| % of the employed in the community | -0.54 | 1.38 | 0.698 | (-3.25, 2.18) |
| % Hispanic in the community | 0.20 | 0.30 | 0.496 | (-0.38, 0.79) |
| % single in the community | 0.36 | 0.58 | 0.539 | (-0.78, 1.50) |
| % of the poor in the community | -0.95 | 0.94 | 0.312 | (-2.79, 0.89) |
| Social Security income | -0.03 | 0.03 | 0.369 | (-0.10, 0.04) |
| Median household income | 0.00 | 0.00 | 0.237 | (0.00, 0.01) |
| % with education less than high school | 0.27 | 0.63 | 0.661 | (-0.95, 1.50) |
| % sensory disability among elderly | -1.23 | 0.99 | 0.214 | (-3.17, 0.71) |
| % non-institutionalized elderly with physical disability | -0.32 | 0.77 | 0.679 | (-1.82, 1.18) |
| % people with mental disability in the community | 0.38 | 1.25 | 0.760 | (-2.07, 2.84) |
| % people with self-care disability | -0.24 | 1.53 | 0.875 | (-3.25, 2.76) |
| % people with difficulty going-outside-the-home disability | 0.77 | 0.96 | 0.425 | (-1.11, 2.65) |
| % elderly in an institution | -1.17 | 0.58 | 0.045 | (-2.31, -0.03) |
| Admission type (Ref = Emergency) |  |  |  |  |
| Urgent | 0.02 | 0.08 | 0.798 | (-0.14, 0.19) |
| Elective | 0.31 | 0.09 | 0.001 | (0.13, 0.50) |
| Newborn | -0.52 | 0.64 | 0.417 | (-1.78, 0.74) |
| Diagnosis codes | Included | Included | Included | Included |
| Constant | 3.95 | 1.57 | 0.012 | (0.88, 7.03) |
|  |  |  |  |  |
| Number of observations: 5,267  Log pseudolikelihood: -2,898.14  Pseudo R^2^: 0.08 |  |  |  |  |
|  | | | | |
