# Supplementary material for: Trends in the quality and cost of inpatient surgical procedures in the United States, 2002–2015
Source: PLoS One. 2021 Nov 3;16(11):e0259011. doi: 10.1371/journal.pone.0259011 (PMC8565758; doi:10.1371/journal.pone.0259011)
Supplement: S10 Table — (A) Regression results for cost of CCS 78 colorectal resection on a year indicator. (B) Regression results for quality of CCS 78 colorectal resection on a year indicator. (DOCX) [file pone.0259011.s010.docx]

**S17 Table.** Regression Results for Cost and Quality of CCS 78 Colorectal Resection on a Year Indicator

S17A Table. Regression results for cost of CCS 78 colorectal resection on a year indicator

| Cost of CCS 78 | Coefficient | Robust standard error | P-value | 95% confidence interval |
| --- | --- | --- | --- | --- |
| Year 2015 | -1.27 | 0.30 | < 0.001 | (-1.85, -0.68) |
| Age | 0.03 | 0.01 | 0.002 | (0.01, 0.05) |
| Race (Ref = White) |  |  |  |  |
| Black | 1.69 | 0.35 | < 0.001 | (1.01, 2.37) |
| Asian | -0.02 | 0.57 | 0.971 | (-1.13, 1.09) |
| Hispanic | 1.40 | 0.82 | 0.089 | (-0.21, 3.02) |
| Female | -0.39 | 0.14 | 0.004 | (-0.65, -0.12) |
| Number of Charlson-Deyo comorbidity (Ref = 0) |  |  |  |  |
| 1 | 0.86 | 0.18 | < 0.001 | (0.49, 1.22) |
| 2 | 1.11 | 0.22 | < 0.001 | (0.67, 1.54) |
| 3 | 2.18 | 0.27 | < 0.001 | (1.66, 2.71) |
| 4 | 1.83 | 0.39 | < 0.001 | (1.06, 2.59) |
| 5 | 1.27 | 0.96 | 0.188 | (-0.62, 3.16) |
| Teaching hospital | 0.17 | 0.31 | 0.594 | (-0.44, 0.77) |
| Transferred from other hospitals | 3.43 | 0.70 | < 0.001 | (2.05, 4.81) |
| Transferred to other hospitals | 2.51 | 0.49 | < 0.001 | (1.54, 3.47) |
| Social Characteristics |  |  |  |  |
| % urban in the community | 0.39 | 0.33 | 0.245 | (-0.27, 1.04) |
| % of the employed in the community | -10.18 | 4.29 | 0.018 | (-18.59, -1.76) |
| % Hispanic in the community | 4.71 | 1.28 | < 0.001 | (2.21, 7.21) |
| % single in the community | 3.60 | 1.92 | 0.060 | (-0.16, 7.36) |
| % of the poor in the community | -2.26 | 2.54 | 0.373 | (-7.24, 2.71) |
| Social Security income | -0.03 | 0.11 | 0.753 | (-0.24, 0.18) |
| Median household income | 0.03 | 0.01 | 0.010 | (0.01, 0.05) |
| % with education less than high school | -0.02 | 1.94 | 0.990 | (-3.84, 3.79) |
| % sensory disability among elderly | -0.27 | 2.32 | 0.906 | (-4.82, 4.27) |
| % non-institutionalized elderly with physical disability | 3.20 | 1.77 | 0.070 | (-0.27, 6.67) |
| % people with mental disability in the community | 2.42 | 2.86 | 0.398 | (-3.19, 8.02) |
| % people with self-care disability | 0.68 | 3.33 | 0.839 | (-5.85, 7.20) |
| % people with difficulty going-outside-the-home disability | -1.15 | 2.19 | 0.598 | (-5.44, 3.13) |
| % elderly in an institution | -3.27 | 1.30 | 0.012 | (-5.82, -0.71) |
| Admission type (Ref = Emergency) |  |  |  |  |
| Urgent | -1.35 | 0.30 | < 0.001 | (-1.94, -0.76) |
| Elective | -3.83 | 0.20 | < 0.001 | (-4.22, -3.43) |
| Newborn | -5.17 | 1.43 | < 0.001 | (-7.97, -2.38) |
| Diagnosis codes | Included | Included | Included | Included |
| Constant | 36.31 | 4.57 | < 0.001 | (27.35, 45.28) |
|  |  |  |  |  |
| Number of observations: 29,975  R-squared: 0.08  Root MSE: 10.38 | | | | |

S17B Table. Regression results for quality of CCS 78 colorectal resection on a year indicator

| Quality of CCS 78 | Coefficient | Robust standard error | P-value | 95% confidence interval |
| --- | --- | --- | --- | --- |
| Year 2015 | 0.01 | 0.03 | 0.756 | (-0.06, 0.08) |
| Age | -0.03 | 0.00 | < 0.001 | (-0.03, -0.02) |
| Race (Ref = White) |  |  |  |  |
| Black | -0.16 | 0.06 | 0.008 | (-0.28, -0.04) |
| Asian | -0.03 | 0.10 | 0.767 | (-0.23, 0.17) |
| Hispanic | 0.07 | 0.15 | 0.641 | (-0.22, 0.36) |
| Female | 0.13 | 0.03 | < 0.001 | (0.07, 0.20) |
| Number of Charlson-Deyo comorbidity (Ref = 0) |  |  |  |  |
| 1 | -0.28 | 0.04 | < 0.001 | (-0.37, -0.20) |
| 2 | -0.51 | 0.05 | < 0.001 | (-0.61, -0.41) |
| 3 | -0.77 | 0.06 | < 0.001 | (-0.89, -0.65) |
| 4 | -0.83 | 0.10 | < 0.001 | (-1.02, -0.63) |
| 5 | -1.19 | 0.23 | < 0.001 | (-1.64, -0.75) |
| Teaching hospital | -0.01 | 0.03 | 0.752 | (-0.06, 0.05) |
| Transferred from other hospitals | -0.59 | 0.10 | < 0.001 | (-0.78, -0.40) |
| Transferred to other hospitals | 0.04 | 0.10 | 0.677 | (-0.15, 0.23) |
| Social Characteristics |  |  |  |  |
| % urban in the community | -0.13 | 0.06 | 0.039 | (-0.26, -0.01) |
| % of the employed in the community | 0.02 | 0.70 | 0.975 | (-1.36, 1.40) |
| % Hispanic in the community | 0.10 | 0.14 | 0.452 | (-0.17, 0.38) |
| % single in the community | 0.41 | 0.30 | 0.168 | (-0.17, 1.00) |
| % of the poor in the community | -0.14 | 0.43 | 0.739 | (-0.99, 0.70) |
| Social Security income | 0.02 | 0.02 | 0.226 | (-0.01, 0.06) |
| Median household income | 0.00 | 0.00 | 0.012 | (0.00, 0.01) |
| % with education less than high school | -0.40 | 0.30 | 0.175 | (-0.98, 0.18) |
| % sensory disability among elderly | 0.21 | 0.46 | 0.652 | (-0.69, 1.11) |
| % non-institutionalized elderly with physical disability | 0.03 | 0.38 | 0.934 | (-0.71, 0.77) |
| % people with mental disability in the community | 0.25 | 0.57 | 0.661 | (-0.87, 1.37) |
| % people with self-care disability | -0.50 | 0.69 | 0.473 | (-1.86, 0.86) |
| % people with difficulty going-outside-the-home disability | 0.60 | 0.44 | 0.179 | (-0.27, 1.47) |
| % elderly in an institution | -0.53 | 0.28 | 0.054 | (-1.08, 0.01) |
| Admission type (Ref = Emergency) |  |  |  |  |
| Urgent | 0.25 | 0.05 | < 0.001 | (0.16, 0.35) |
| Elective | 0.61 | 0.04 | < 0.001 | (0.54, 0.69) |
| Newborn | -0.19 | 0.22 | 0.407 | (-0.63, 0.25) |
| Diagnosis codes | Included | Included | Included | Included |
| Constant | 2.60 | 0.81 | 0.001 | (1.02, 4.19) |
|  |  |  |  |  |
| Number of observations: 29,975  Log pseudolikelihood: -13,667.64  Pseudo R^2^: 0.07 | | | | |
