# Supplementary material for: Trends in the quality and cost of inpatient surgical procedures in the United States, 2002–2015
Source: PLoS One. 2021 Nov 3;16(11):e0259011. doi: 10.1371/journal.pone.0259011 (PMC8565758; doi:10.1371/journal.pone.0259011)
Supplement: S11 Table — (A) Regression results for cost of CCS 89 exploratory laparotomy on a year indicator. (B) Regression results for quality of CCS 89 exploratory laparotomy on a year indicator. (DOCX) [file pone.0259011.s011.docx]

**S18 Table.** Regression Results for Cost and Quality of CCS 89 Exploratory Laparotomy on a Year Indicator

S18A Table. Regression results for cost of CCS 89 exploratory laparotomy on a year indicator

| Cost of CCS 89 | Coefficient | Robust standard error | P-value | 95% confidence interval |
| --- | --- | --- | --- | --- |
| Year 2015 | -1.16 | 1.95 | 0.551 | (-4.98, 2.66) |
| Age | -0.04 | 0.09 | 0.659 | (-0.23, 0.14) |
| Race (Ref = White) |  |  |  |  |
| Black | 4.37 | 3.77 | 0.246 | (-3.02, 11.77) |
| Asian | 9.92 | 7.89 | 0.209 | (-5.57, 25.41) |
| Hispanic | -7.21 | 4.03 | 0.074 | (-15.12, 0.71) |
| Female | -3.98 | 1.50 | 0.008 | (-6.93, -1.04) |
| Number of Charlson-Deyo comorbidity (Ref = 0) |  |  |  |  |
| 1 | 0.14 | 1.78 | 0.937 | (-3.36, 3.64) |
| 2 | 4.34 | 2.11 | 0.040 | (0.21, 8.48) |
| 3 | 0.42 | 2.00 | 0.833 | (-3.51, 4.35) |
| 4 | 1.70 | 3.00 | 0.571 | (-4.18, 7.58) |
| 5 |  |  |  |  |
| Teaching hospital | 2.43 | 1.28 | 0.057 | (-0.08, 4.94) |
| Transferred from other hospitals | -1.94 | 3.10 | 0.531 | (-8.02, 4.14) |
| Transferred to other hospitals | 4.29 | 3.61 | 0.236 | (-2.81, 11.38) |
| Social Characteristics |  |  |  |  |
| % urban in the community | 0.31 | 2.38 | 0.897 | (-4.36, 4.97) |
| % of the employed in the community | 17.67 | 20.87 | 0.398 | (-23.31, 58.64) |
| % Hispanic in the community | 0.69 | 5.22 | 0.895 | (-9.56, 10.95) |
| % single in the community | 13.52 | 10.53 | 0.199 | (-7.15, 34.19) |
| % of the poor in the community | -0.90 | 16.32 | 0.956 | (-32.95, 31.15) |
| Social Security income | -1.30 | 0.68 | 0.055 | (-2.64, 0.03) |
| Median household income | 0.13 | 0.09 | 0.175 | (-0.06, 0.31) |
| % with education less than high school | -5.63 | 12.54 | 0.654 | (-30.25, 19.00) |
| % sensory disability among elderly | -13.93 | 20.50 | 0.497 | (-54.18, 26.32) |
| % non-institutionalized elderly with physical disability | 16.62 | 14.53 | 0.253 | (-11.90, 45.15) |
| % people with mental disability in the community | -19.18 | 25.37 | 0.450 | (-68.99, 30.62) |
| % people with self-care disability | -31.04 | 27.24 | 0.255 | (-84.51, 22.44) |
| % people with difficulty going-outside-the-home disability | 22.13 | 17.28 | 0.201 | (-11.79, 56.06) |
| % elderly in an institution | -4.13 | 11.57 | 0.721 | (-26.85, 18.59) |
| Admission type (Ref = Emergency) |  |  |  |  |
| Urgent | 1.42 | 1.79 | 0.426 | (-2.08, 4.93) |
| Elective | -4.88 | 1.60 | 0.002 | (-8.02, -1.73) |
| Newborn | 8.20 | 9.30 | 0.379 | (-10.07, 26.46) |
| Diagnosis codes | Included | Included | Included | Included |
| Constant | 17.54 | 24.25 | 0.470 | (-30.07, 65.16) |
|  |  |  |  |  |
| Number of observations: 1,018  R-squared: 0.16  Root MSE: 19.52 | | | | |

S18B Table. Regression results for quality of CCS 89 exploratory laparotomy on a year indicator

| Quality of CCS 89 | Coefficient | Robust standard error | P-value | 95% confidence interval |
| --- | --- | --- | --- | --- |
| Year 2015 | 0.10 | 0.20 | 0.602 | (-0.28, 0.49) |
| Age | -0.04 | 0.01 | < 0.001 | (-0.06, -0.02) |
| Race (Ref = White) |  |  |  |  |
| Black | -0.16 | 0.28 | 0.567 | (-0.70, 0.39) |
| Asian | 0.13 | 0.46 | 0.772 | (-0.77, 1.03) |
| Hispanic | -0.87 | 0.72 | 0.228 | (-2.28, 0.54) |
| Female | 0.11 | 0.17 | 0.534 | (-0.23, 0.44) |
| Number of Charlson-Deyo comorbidity (Ref = 0) |  |  |  |  |
| 1 | -0.71 | 0.21 | 0.001 | (-1.13, -0.29) |
| 2 | -0.80 | 0.24 | 0.001 | (-1.28, -0.33) |
| 3 | -1.31 | 0.32 | < 0.001 | (-1.93, -0.68) |
| 4 | -1.12 | 0.48 | 0.021 | (-2.06, -0.17) |
| 5 |  |  |  |  |
| Teaching hospital | -0.35 | 0.14 | 0.012 | (-0.63, -0.08) |
| Transferred from other hospitals | -0.52 | 0.36 | 0.156 | (-1.23, 0.20) |
| Transferred to other hospitals | 1.33 | 0.63 | 0.034 | (0.10, 2.56) |
| Social Characteristics |  |  |  |  |
| % urban in the community | -0.39 | 0.29 | 0.186 | (-0.96, 0.19) |
| % of the employed in the community | -7.14 | 3.46 | 0.039 | (-13.92, -0.35) |
| % Hispanic in the community | 0.40 | 0.70 | 0.564 | (-0.96, 1.77) |
| % single in the community | 2.25 | 1.37 | 0.102 | (-0.44, 4.94) |
| % of the poor in the community | -3.52 | 2.15 | 0.101 | (-7.74, 0.69) |
| Social Security income | -0.01 | 0.08 | 0.891 | (-0.17, 0.15) |
| Median household income | 0.01 | 0.01 | 0.266 | (-0.01, 0.03) |
| % with education less than high school | -0.36 | 1.60 | 0.824 | (-3.49, 2.78) |
| % sensory disability among elderly | 0.53 | 2.18 | 0.807 | (-3.74, 4.80) |
| % non-institutionalized elderly with physical disability | -1.53 | 2.04 | 0.452 | (-5.54, 2.47) |
| % people with mental disability in the community | 2.53 | 3.13 | 0.420 | (-3.61, 8.67) |
| % people with self-care disability | 2.12 | 3.87 | 0.583 | (-5.47, 9.72) |
| % people with difficulty going-outside-the-home disability | -0.41 | 1.92 | 0.833 | (-4.17, 3.36) |
| % elderly in an institution | 1.96 | 1.56 | 0.210 | (-1.10, 5.02) |
| Admission type (Ref = Emergency) |  |  |  |  |
| Urgent | 0.03 | 0.21 | 0.901 | (-0.38, 0.43) |
| Elective | 1.13 | 0.23 | < 0.001 | (0.69, 1.57) |
| Newborn | 0.35 | 0.88 | 0.685 | (-1.36, 2.07) |
| Diagnosis codes | Included | Included | Included | Included |
| Constant | 10.13 | 3.83 | 0.008 | (2.61, 17.64) |
|  |  |  |  |  |
| Number of observations: 1,018  Log pseudolikelihood: -549.25  Pseudo R^2^: 0.22 | | | | |
