# Supplementary material for: Trends in the quality and cost of inpatient surgical procedures in the United States, 2002–2015
Source: PLoS One. 2021 Nov 3;16(11):e0259011. doi: 10.1371/journal.pone.0259011 (PMC8565758; doi:10.1371/journal.pone.0259011)
Supplement: S12 Table — (A) Regression results for cost of CCS 90 excision, lysis peritoneal adhesions on a year indicator. (B) Regression results for quality of CCS 90 excision, lysis peritoneal adhesions on a year indicator. (DOCX) [file pone.0259011.s012.docx]

**S19 Table.** Regression Results for Cost and Quality of CCS 90 Excision, Lysis Peritoneal Adhesions on a Year Indicator

S19A Table. Regression results for cost of CCS 90 excision, lysis peritoneal adhesions on a year indicator

| Cost of CCS 90 | Coefficient | Robust standard error | P-value | 95% confidence interval |
| --- | --- | --- | --- | --- |
| Year 2015 | -1.16 | 1.95 | 0.551 | (-4.98, 2.66) |
| Age | -0.04 | 0.09 | 0.659 | (-0.23, 0.14) |
| Race (Ref = White) |  |  |  |  |
| Black | 4.37 | 3.77 | 0.246 | (-3.02, 11.77) |
| Asian | 9.92 | 7.89 | 0.209 | (-5.57, 25.41) |
| Hispanic | -7.21 | 4.03 | 0.074 | (-15.12, 0.71) |
| Female | -3.98 | 1.50 | 0.008 | (-6.93, -1.04) |
| Number of Charlson-Deyo comorbidity (Ref = 0) |  |  |  |  |
| 1 | 0.14 | 1.78 | 0.937 | (-3.36, 3.64) |
| 2 | 4.34 | 2.11 | 0.040 | (0.21, 8.48) |
| 3 | 0.42 | 2.00 | 0.833 | (-3.51, 4.35) |
| 4 | 1.70 | 3.00 | 0.571 | (-4.18, 7.58) |
| Teaching hospital | 2.43 | 1.28 | 0.057 | (-0.08, 4.94) |
| Transferred from other hospitals | -1.94 | 3.10 | 0.531 | (-8.02, 4.14) |
| Transferred to other hospitals | 4.29 | 3.61 | 0.236 | (-2.81, 11.38) |
| Social Characteristics |  |  |  |  |
| % urban in the community | 0.31 | 2.38 | 0.897 | (-4.36, 4.97) |
| % of the employed in the community | 17.67 | 20.87 | 0.398 | (-23.31, 58.64) |
| % Hispanic in the community | 0.69 | 5.22 | 0.895 | (-9.56, 10.95) |
| % single in the community | 13.52 | 10.53 | 0.199 | (-7.15, 34.19) |
| % of the poor in the community | -0.90 | 16.32 | 0.956 | (-32.95, 31.15) |
| Social Security income | -1.30 | 0.68 | 0.055 | (-2.64, 0.03) |
| Median household income | 0.13 | 0.09 | 0.175 | (-0.06, 0.31) |
| % with education less than high school | -5.63 | 12.54 | 0.654 | (-30.25, 19.00) |
| % sensory disability among elderly | -13.93 | 20.50 | 0.497 | (-54.18, 26.32) |
| % non-institutionalized elderly with physical disability | 16.62 | 14.53 | 0.253 | (-11.90, 45.15) |
| % people with mental disability in the community | -19.18 | 25.37 | 0.450 | (-68.99, 30.62) |
| % people with self-care disability | -31.04 | 27.24 | 0.255 | (-84.51, 22.44) |
| % people with difficulty going-outside-the-home disability | 22.13 | 17.28 | 0.201 | (-11.79, 56.06) |
| % elderly in an institution | -4.13 | 11.57 | 0.721 | (-26.85, 18.59) |
| Admission type (Ref = Emergency) |  |  |  |  |
| Urgent | 0.28 | 0.71 | 0.692 | (-1.12, 1.68) |
| Elective | -4.41 | 0.61 | < 0.001 | (-5.61, -3.21) |
| Newborn |  |  |  |  |
| Diagnosis codes | Included | Included | Included | Included |
| Constant | -1.91 | 10.18 | 0.851 | (-21.87, 18.05) |
|  |  |  |  |  |
| Number of observations: 5,824  R-squared: 0.07  Root MSE: 15.58 | | | | |

S19B Table. Regression results for quality of CCS 90 excision, lysis peritoneal adhesions on a year indicator

| Quality of CCS 90 | Coefficient | Robust standard error | P-value | 95% confidence interval |
| --- | --- | --- | --- | --- |
| Year 2015 | -0.03 | 0.08 | 0.673 | (-0.18, 0.12) |
| Age | -0.03 | 0.00 | < 0.001 | (-0.04, -0.02) |
| Race (Ref = White) |  |  |  |  |
| Black | -0.11 | 0.13 | 0.375 | (-0.36, 0.14) |
| Asian | -0.20 | 0.23 | 0.389 | (-0.64, 0.25) |
| Hispanic | 0.22 | 0.30 | 0.478 | (-0.38, 0.81) |
| Female | 0.17 | 0.07 | 0.017 | (0.03, 0.31) |
| Number of Charlson-Deyo comorbidity (Ref = 0) |  |  |  |  |
| 1 | -0.34 | 0.08 | < 0.001 | (-0.49, -0.18) |
| 2 | -0.60 | 0.10 | < 0.001 | (-0.80, -0.40) |
| 3 | -0.62 | 0.17 | < 0.001 | (-0.95, -0.29) |
| 4 | -0.75 | 0.39 | 0.051 | (-1.51, 0.00) |
| Teaching hospital | 0.04 | 0.06 | 0.517 | (-0.08, 0.17) |
| Transferred from other hospitals | 0.37 | 0.21 | 0.069 | (-0.03, 0.78) |
| Transferred to other hospitals | 0.02 | 0.24 | 0.945 | (-0.46, 0.49) |
| Social Characteristics |  |  |  |  |
| % urban in the community | 0.16 | 0.13 | 0.234 | (-0.10, 0.42) |
| % of the employed in the community | 0.46 | 1.40 | 0.744 | (-2.28, 3.20) |
| % Hispanic in the community | 0.08 | 0.30 | 0.805 | (-0.52, 0.67) |
| % single in the community | 0.55 | 0.62 | 0.378 | (-0.67, 1.76) |
| % of the poor in the community | -1.17 | 0.95 | 0.216 | (-3.03, 0.69) |
| Social Security income | -0.03 | 0.04 | 0.400 | (-0.10, 0.04) |
| Median household income | 0.00 | 0.00 | 0.875 | (-0.01, 0.01) |
| % with education less than high school | -0.44 | 0.64 | 0.487 | (-1.69, 0.80) |
| % sensory disability among elderly | 0.88 | 1.12 | 0.432 | (-1.32, 3.08) |
| % non-institutionalized elderly with physical disability | 0.28 | 0.95 | 0.768 | (-1.58, 2.14) |
| % people with mental disability in the community | 0.44 | 1.33 | 0.738 | (-2.16, 3.04) |
| % people with self-care disability | -2.35 | 1.50 | 0.117 | (-5.28, 0.59) |
| % people with difficulty going-outside-the-home disability | -0.45 | 0.89 | 0.612 | (-2.20, 1.30) |
| % elderly in an institution | -1.05 | 0.63 | 0.096 | (-2.29, 0.19) |
| Admission type (Ref = Emergency) |  |  |  |  |
| Urgent | 0.00 | 0.09 | 0.977 | (-0.17, 0.18) |
| Elective | 0.40 | 0.10 | < 0.001 | (0.19, 0.60) |
| Newborn |  |  |  |  |
| Diagnosis codes | Included | Included | Included | Included |
| Constant | 3.10 | 1.58 | 0.049 | (0.01, 6.18) |
|  |  |  |  |  |
| Number of observations: 5,824  Log pseudolikelihood: -2,697.86  Pseudo R^2^: 0.05 | | | | |
