# Supplementary material for: Trends in the quality and cost of inpatient surgical procedures in the United States, 2002–2015
Source: PLoS One. 2021 Nov 3;16(11):e0259011. doi: 10.1371/journal.pone.0259011 (PMC8565758; doi:10.1371/journal.pone.0259011)
Supplement: S13 Table — (A) Regression results for cost of CCS169 debridement of wound on a year indicator. (B) Regression results for quality of CCS169 debridement of wound on a year indicator. (DOCX) [file pone.0259011.s013.docx]

**S20 Table.** Regression Results for Cost and Quality of CCS169 Debridement of Wound on a Year Indicator

S20A Table. Regression results for cost of CCS169 debridement of wound on a year indicator

| Cost of CCS169 | Coefficient | Robust standard error | P-value | 95% confidence interval |
| --- | --- | --- | --- | --- |
| Year 2015 | -3.72 | 0.38 | < 0.001 | (-4.47, -2.98) |
| Age | -0.09 | 0.01 | < 0.001 | (-0.11, -0.07) |
| Race (Ref = White) |  |  |  |  |
| Black | 1.34 | 0.33 | < 0.001 | (0.71, 1.98) |
| Asian | 2.71 | 1.04 | 0.009 | (0.68, 4.75) |
| Hispanic | -0.59 | 0.68 | 0.387 | (-1.92, 0.74) |
| Female | -0.21 | 0.20 | 0.300 | (-0.61, 0.19) |
| Number of Charlson-Deyo comorbidity (Ref = 0) |  |  |  |  |
| 1 | 0.03 | 0.25 | 0.917 | (-0.46, 0.51) |
| 2 | 0.20 | 0.26 | 0.448 | (-0.31, 0.71) |
| 3 | 0.44 | 0.37 | 0.234 | (-0.28, 1.16) |
| 4 | 0.12 | 0.64 | 0.854 | (-1.13, 1.36) |
| Teaching hospital | -2.48 | 1.43 |  | (-5.28, 0.32) |
| Transferred from other hospitals | 2.91 | 0.42 | < 0.001 | (2.09, 3.72) |
| Transferred to other hospitals | 4.44 | 0.99 | < 0.001 | (2.51, 6.37) |
| Social Characteristics | 0.85 | 0.46 | 0.063 | (-0.05, 1.75) |
| % urban in the community |  |  |  |  |
| % of the employed in the community | -0.72 | 0.44 | 0.103 | (-1.58, 0.14) |
| % Hispanic in the community | 6.46 | 4.86 | 0.184 | (-3.06, 15.98) |
| % single in the community | 5.29 | 1.19 | < 0.001 | (2.95, 7.63) |
| % of the poor in the community | 9.07 | 2.29 | < 0.001 | (4.57, 13.57) |
| Social Security income | 5.70 | 3.21 | 0.076 | (-0.59, 11.99) |
| Median household income | -0.10 | 0.15 | 0.485 | (-0.39, 0.18) |
| % with education less than high school | 0.11 | 0.02 | < 0.001 | (0.07, 0.14) |
| % sensory disability among elderly | 0.47 | 2.47 | 0.848 | (-4.37, 5.31) |
| % non-institutionalized elderly with physical disability | -1.12 | 2.86 | 0.696 | (-6.74, 4.50) |
| % people with mental disability in the community | -3.25 | 2.64 | 0.219 | (-8.42, 1.93) |
| % people with self-care disability | 3.44 | 4.02 | 0.393 | (-4.45, 11.33) |
| % people with difficulty going-outside-the-home disability | 3.60 | 6.52 | 0.581 | (-9.18, 16.38) |
| % elderly in an institution | -0.58 | 3.53 | 0.870 | (-7.50, 6.35) |
| Admission type (Ref = Emergency) |  |  |  |  |
| Urgent | -0.45 | 0.32 | 0.170 | (-1.08, 0.19) |
| Elective | -0.85 | 0.29 | 0.004 | (-1.43, -0.28) |
| Newborn | 7.29 | 3.95 | 0.065 | (-0.46, 15.04) |
| Diagnosis codes | Included | Included | Included | Included |
| Constant | 11.56 | 5.27 | 0.028 | (1.23, 21.89) |
|  |  |  |  |  |
| Number of observations: 15,355  R-squared: 0.12  Root MSE: 10.81 | | | | |

S20B Table. Regression results for quality of CCS169 debridement of wound on a year indicator

| Quality of CCS169 | Coefficient | Robust standard error | P-value | 95% confidence interval |
| --- | --- | --- | --- | --- |
| Year 2015 | 0.27 | 0.05 | < 0.001 | (0.18, 0.37) |
| Age | -0.02 | 0.00 | < 0.001 | (-0.03, -0.02) |
| Race (Ref = White) |  |  |  |  |
| Black | 0.01 | 0.06 | 0.838 | (-0.11, 0.13) |
| Asian | -0.22 | 0.12 | 0.080 | (-0.46, 0.03) |
| Hispanic | -0.11 | 0.14 | 0.415 | (-0.38, 0.16) |
| Female | -0.04 | 0.04 | 0.368 | (-0.11, 0.04) |
| Number of Charlson-Deyo comorbidity (Ref = 0) |  |  |  |  |
| 1 | -0.28 | 0.05 | < 0.001 | (-0.38, -0.18) |
| 2 | -0.42 | 0.06 | < 0.001 | (-0.54, -0.31) |
| 3 | -0.63 | 0.07 | < 0.001 | (-0.78, -0.49) |
| 4 | -0.84 | 0.15 | < 0.001 | (-1.13, -0.55) |
| Teaching hospital | -0.92 | 0.41 |  | (-1.72, -0.12) |
| Transferred from other hospitals | -0.05 | 0.04 | 0.203 | (-0.12, 0.02) |
| Transferred to other hospitals | -0.35 | 0.12 | 0.004 | (-0.60, -0.11) |
| Social Characteristics | 0.37 | 0.10 | < 0.001 | (0.17, 0.56) |
| % urban in the community |  |  |  |  |
| % of the employed in the community | -0.04 | 0.08 | 0.590 | (-0.20, 0.11) |
| % Hispanic in the community | 0.06 | 0.86 | 0.943 | (-1.62, 1.74) |
| % single in the community | 0.44 | 0.16 | 0.005 | (0.13, 0.75) |
| % of the poor in the community | 0.08 | 0.35 | 0.822 | (-0.60, 0.76) |
| Social Security income | -0.83 | 0.50 | 0.097 | (-1.82, 0.15) |
| Median household income | 0.02 | 0.02 | 0.437 | (-0.02, 0.06) |
| % with education less than high school | 0.00 | 0.00 | 0.340 | (-0.01, 0.00) |
| % sensory disability among elderly | -0.38 | 0.34 | 0.263 | (-1.05, 0.29) |
| % non-institutionalized elderly with physical disability | 0.67 | 0.56 | 0.232 | (-0.43, 1.77) |
| % people with mental disability in the community | 0.19 | 0.48 | 0.687 | (-0.75, 1.13) |
| % people with self-care disability | -1.09 | 0.63 | 0.084 | (-2.33, 0.15) |
| % people with difficulty going-outside-the-home disability | 1.36 | 0.75 | 0.069 | (-0.11, 2.84) |
| % elderly in an institution | -1.35 | 0.55 | 0.014 | (-2.43, -0.27) |
| Admission type (Ref = Emergency) |  |  |  |  |
| Urgent | 0.17 | 0.05 | 0.001 | (0.07, 0.27) |
| Elective | 0.39 | 0.06 | < 0.001 | (0.28, 0.50) |
| Newborn | -0.45 | 0.25 | 0.073 | (-0.94, 0.04) |
| Diagnosis codes | Included | Included | Included | Included |
| Constant | 3.35 | 0.97 | 0.001 | (1.45, 5.24) |
|  |  |  |  |  |
| Number of observations: 15,355  Log pseudolikelihood: -8,067.83  Pseudo R^2^: 0.06 | | | | |
