# Supplementary material for: Trends in the quality and cost of inpatient surgical procedures in the United States, 2002–2015
Source: PLoS One. 2021 Nov 3;16(11):e0259011. doi: 10.1371/journal.pone.0259011 (PMC8565758; doi:10.1371/journal.pone.0259011)
Supplement: S1 Fig — (PDF) [file pone.0259011.s014.pdf]

S2 Fig. Trends of Unadjusted and Adjusted Quality for Each Surgical Category, 2002-2015

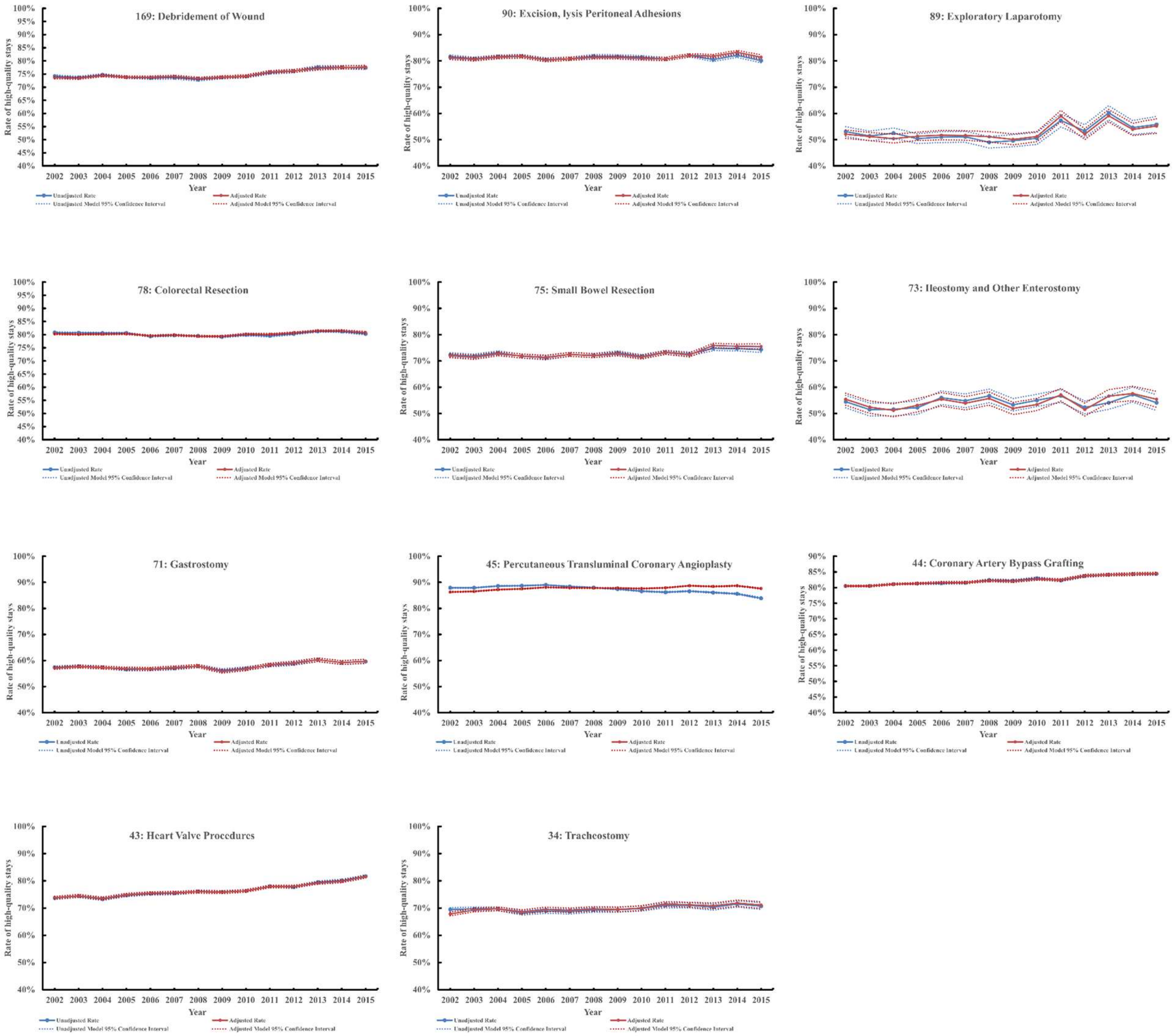

Notes: This figure shows predicted rates of "high-quality stays" and associated confidence intervals from multivariate logistic regressions, in which indicators for 30-day survival without readmissions for patients are the dependent variables. Unadjusted and adjusted models are regressed on year indicators at the individual level. The adjusted regressions are controlled for age, gender, race/ethnicity, the Charlson comorbidities, area sociodemographic, teaching hospital indicators, and inpatient stay characteristics, such as diagnosis codes and inpatient admission types.
