# Supplementary material for: Trends in the quality and cost of inpatient surgical procedures in the United States, 2002–2015
Source: PLoS One. 2021 Nov 3;16(11):e0259011. doi: 10.1371/journal.pone.0259011 (PMC8565758; doi:10.1371/journal.pone.0259011)
Supplement: S2 Fig — (PDF) [file pone.0259011.s015.pdf]

S3 Fig. Trends of Unadjusted and Adjusted Cost for Each Surgical Category, 2002-2015

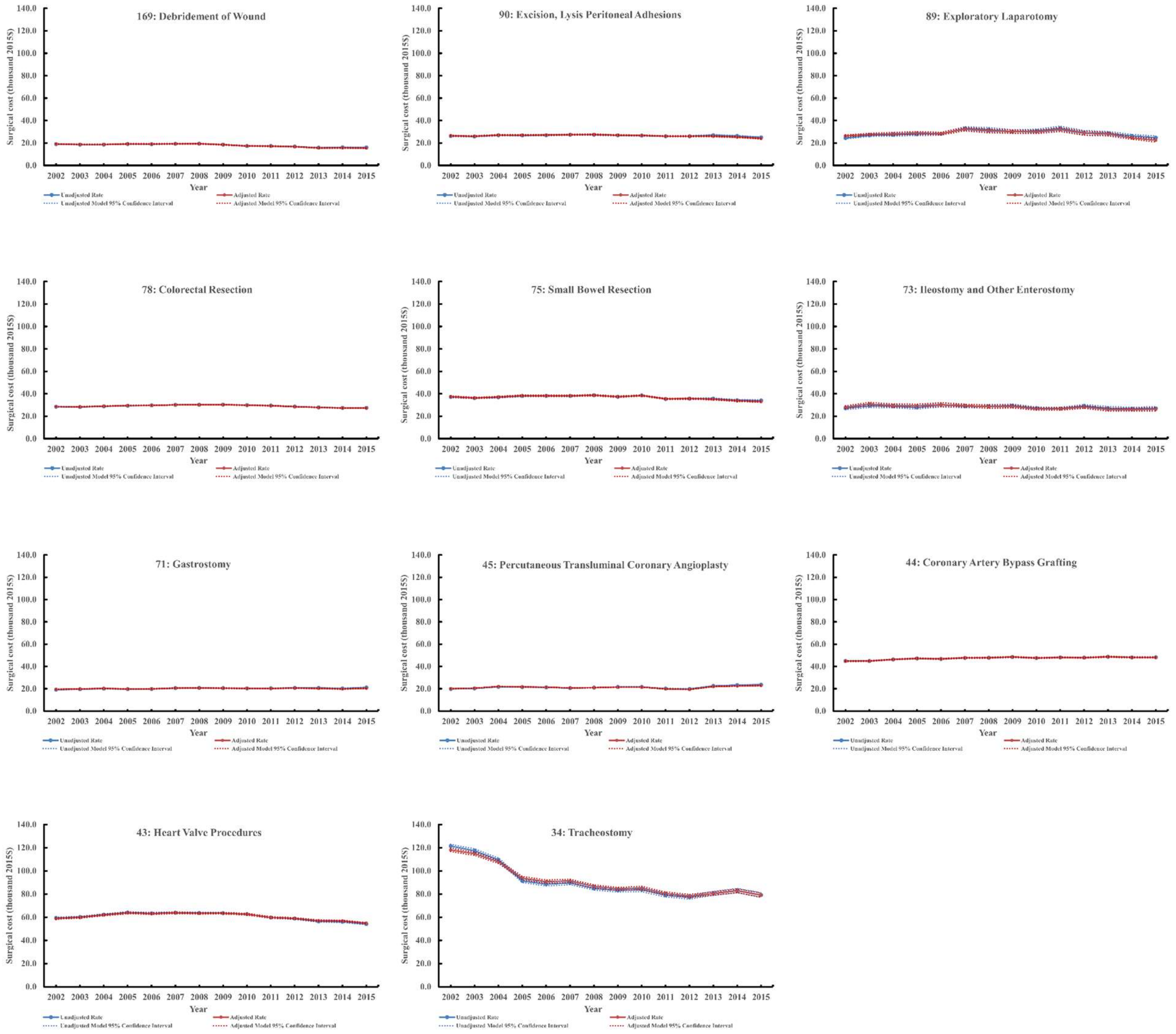

Notes: This figure shows unadjusted and predicted costs and associated standard errors from ordinary least squares regressions, in which the sum of inpatient and non-institutional costs is the dependent variable. Unadjusted and adjusted models are regressed on year indicators at the individual level. The adjusted regressions are controlled for age, gender, race/ethnicity, the Charlson comorbidities, area sociodemographic, teaching hospital indicators, and inpatient stay characteristics, such as diagnosis codes and inpatient admission types.
