# Supplementary material for: Trends in the quality and cost of inpatient surgical procedures in the United States, 2002–2015
Source: PLoS One. 2021 Nov 3;16(11):e0259011. doi: 10.1371/journal.pone.0259011 (PMC8565758; doi:10.1371/journal.pone.0259011)

S4 Fig. Decomposition of Overall Quality Trends into Survival and Avoidance of Unplanned Readmissions

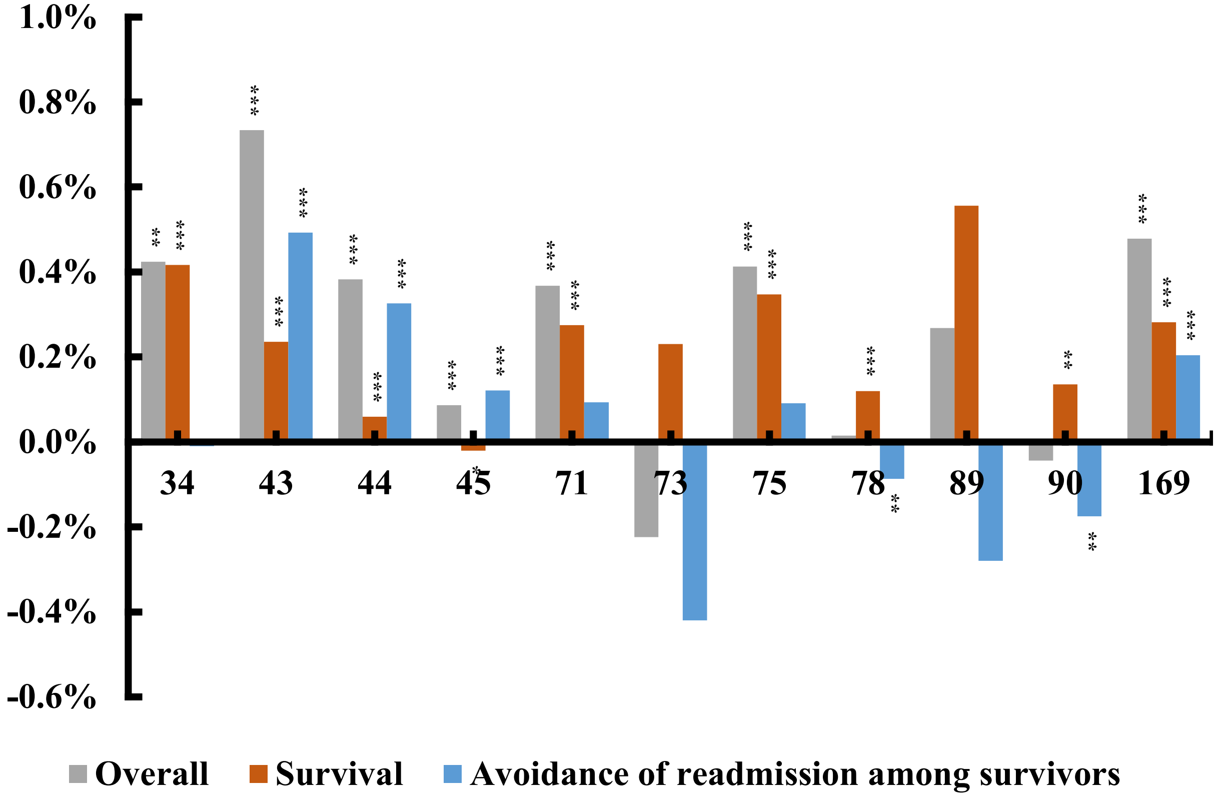

Supplement: S3 Fig — (PDF) [file pone.0259011.s016.pdf]
