# Supplementary material for: Trends in the quality and cost of inpatient surgical procedures in the United States, 2002–2015
Source: PLoS One. 2021 Nov 3;16(11):e0259011. doi: 10.1371/journal.pone.0259011 (PMC8565758; doi:10.1371/journal.pone.0259011)
Supplement: S4 Fig — (PDF) [file pone.0259011.s017.pdf]

S5 Fig. Sensitivity Analysis: Interacting Year Indicator with Patient Age

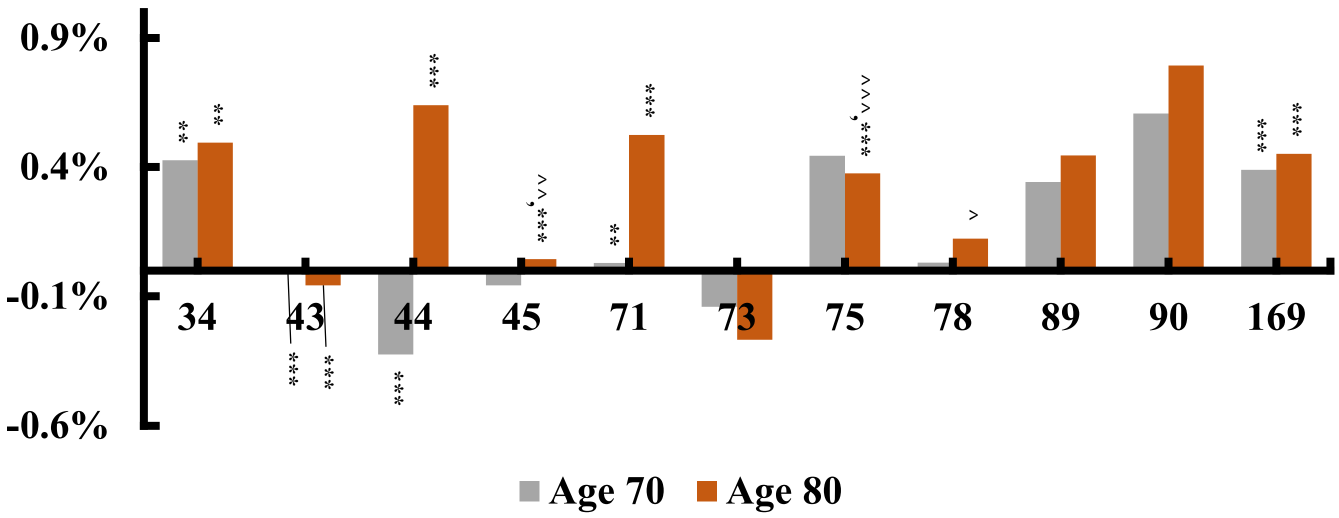

Annualized trends in high-quality (30-day survival without an unplanned readmission) surgeries, 2002-2015, by CCS

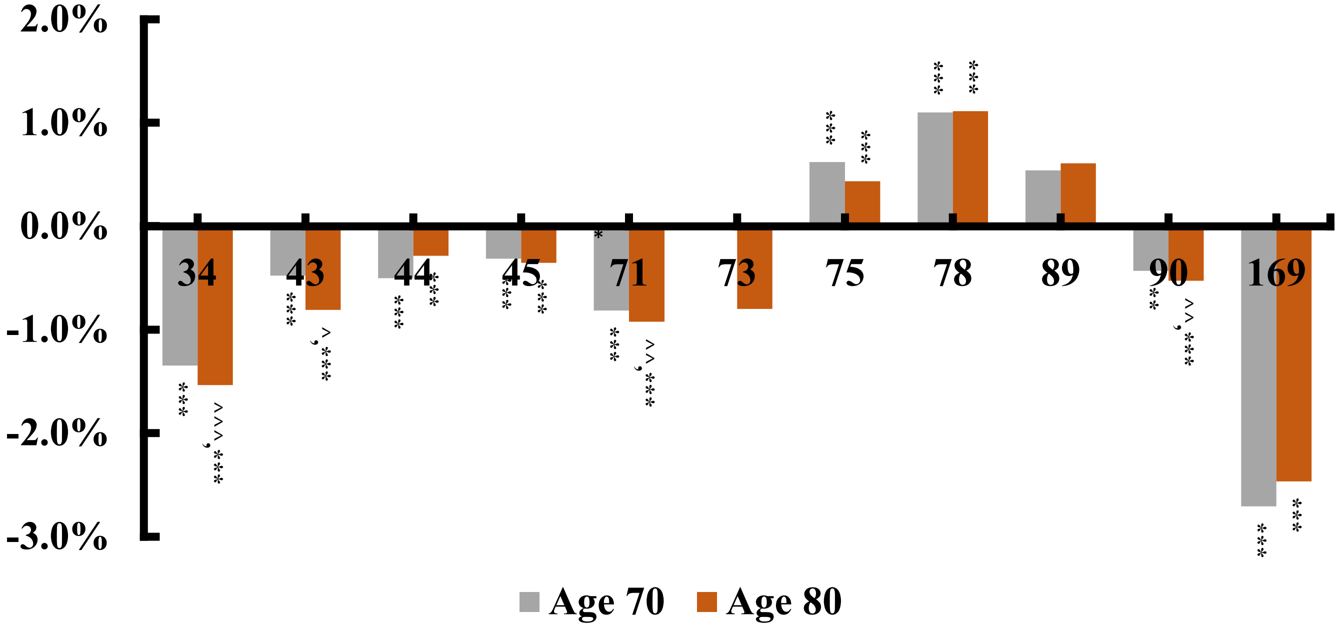

Annualized trends in surgical cost, 2002-2015, by CCS

Notes: ^, ^^ and ^^^ denote significant interactions at 10%, 5% and 1% levels; *, ** and *** denote significant age-specific trends at 10%, 5% and 1% levels.
