# Supplementary material for: Trends in the quality and cost of inpatient surgical procedures in the United States, 2002–2015
Source: PLoS One. 2021 Nov 3;16(11):e0259011. doi: 10.1371/journal.pone.0259011 (PMC8565758; doi:10.1371/journal.pone.0259011)
Supplement: S5 Fig — (A) Unadjusted and adjusted annualized growth rate of surgical quality in sensitivity analysis (quality redefined as rate of surviving 30+ days after discharge without unplanned readmission). (B) Unadjusted and adjusted annualized growth rate of surgical cost (cost redefined as cost per day in hospital). (C) Unadjusted and adjusted annualized change rate of days in hospitals. (PDF) [file pone.0259011.s018.pdf]

S7 Fig. Sensitivity Analysis: Unadjusted and Adjusted Annualized Growth Rate of Quality and Cost with Redefined Quality and Cost Metrics

S7A. Unadjusted and adjusted annualized growth rate of surgical quality in sensitivity analysis (quality redefined as rate of surviving 30+ days after discharge without unplanned readmission)

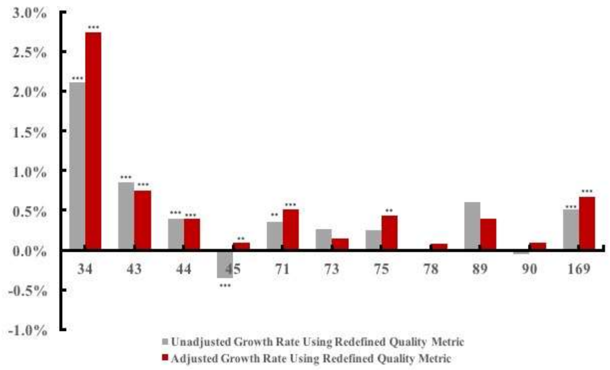

S7B. Unadjusted and adjusted annualized growth rate of surgical cost (cost redefined as cost per day in hospital)

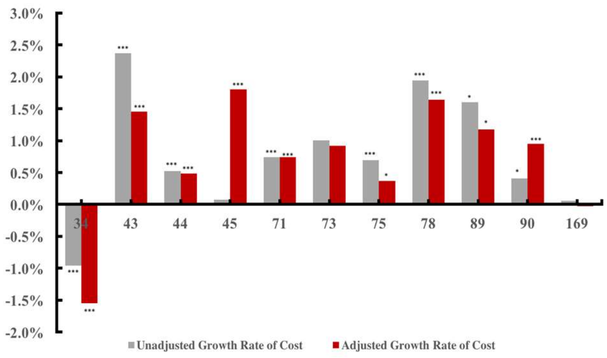

S7C. Unadjusted and adjusted annualized change rate of days in hospitals

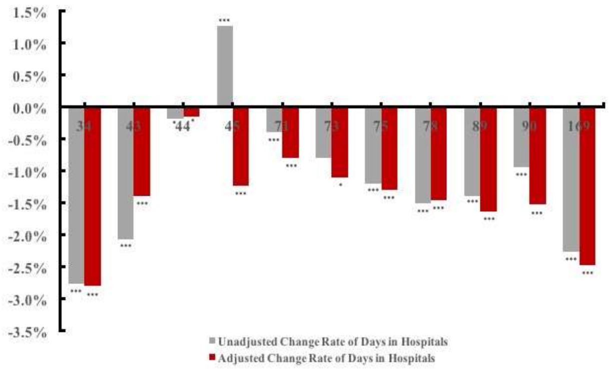

Note: This figure shows annualized growth rates of surgical quality, cost, and days of hospitalizations, calculated based on unadjusted and adjusted quality, cost, and days of hospitalizations in 2002 and 2015. The unadjusted models were regressed on a year indicator (2002 vs 2015) at the individual level. The adjusted quality, cost, and days of hospitalizations were predicted from the adjusted models, which were regressed on a year indicator (2002 vs 2015), demographic information, patient illness severity, hospital characterisitics, and area sociodemographic. In this sensitivity analysis, we redefined quality metric as the rate of 30-day survival without unplanned readmission after hospital discharge, and redefined cost metric as daily cost of hospital stays. * P <0.05; ** P <0.01; *** P <0.001
