# Supplementary material for: Trends in the quality and cost of inpatient surgical procedures in the United States, 2002–2015
Source: PLoS One. 2021 Nov 3;16(11):e0259011. doi: 10.1371/journal.pone.0259011 (PMC8565758; doi:10.1371/journal.pone.0259011)
Supplement: S6 Fig — (PDF) [file pone.0259011.s019.pdf]

**S8 Fig.** Sensitivity Analysis: Adjusted Annualized Growth Rate of Quality and Cost Before and After Adjusting for Extra Risk Factors for Patients with a Heart Attack During Hospitalizations

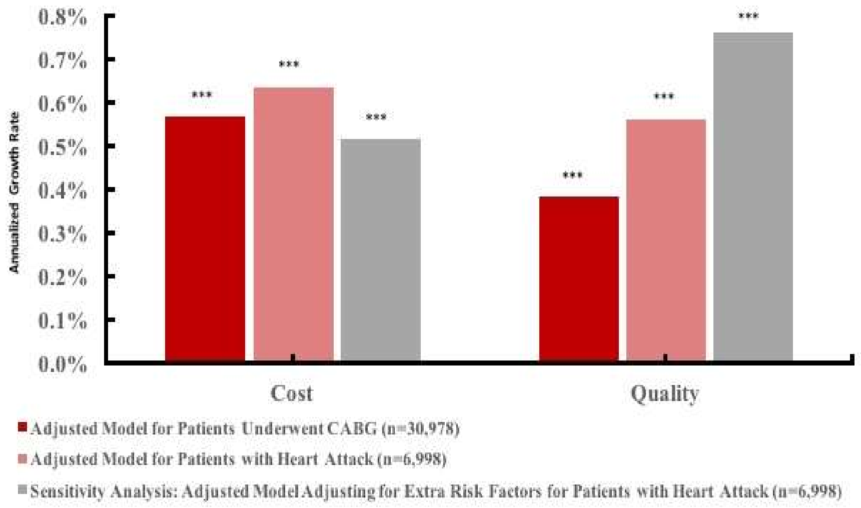


Note: This figure shows annualized growth rate of surgical quality and cost, calculated based on predicted adjusted quality and cost in 2002 and 2015. Patients included in the models were those who underwent CABG in 2002 and 2015. Among all patients underwent CABG (n=30,978), 6,998 patients had a heart attack during their inpatient stays. The adjusted quality and cost were predicted from the adjusted models for CABG, which regressed on a year indicator (2002 vs 2015), demographic information, patient illness severity, hospital characteristics, and area sociodemographic. In sensitivity analysis, we adjusted for specific anatomical locations of the heart attack and predicted likelihood of death during the hospital stays, together with the control variables described above (n=6,998). *** P <0.001
